# Mild hunger elicits attentional desensitization to visual food cues in healthy, non-obese individuals

**DOI:** 10.3389/fpsyg.2024.1441184

**Published:** 2024-09-09

**Authors:** Nicol Schlezingerová, Petra Málková, Martin Kocourek, Petr Telenský

**Affiliations:** ^1^Department of Physiology, Faculty of Science, Charles University, Prague, Czechia; ^2^Third Faculty of Medicine, Charles University, Prague, Czechia

**Keywords:** visual food cues, eye-tracking, EDA, salivary cortisol and alpha-amylase, Food Stroop test, Visual Analog Scale, satiety, hunger

## Abstract

**Introduction:**

Food is a vital human need, and the human visual system is finely tuned to detect and respond to food cues in the environment. The omnipresence of food cues across various settings has been linked to the prevalence of obesity in susceptible populations. However, the influence of the post-prandial state on visual attention to food stimuli remains poorly understood. This study aimed to elucidate how a 12 hour fast affects visual attention to food and non-food stimuli in healthy, non-obese individuals.

**Methods:**

Visual attention was assessed by measuring the total duration of visual fixations on stimuli presented on a computer screen, using a screen-based eye tracker (Tobii X2-60). Participants were divided into two groups: those who had fasted for 12 hours and those tested within two hours after consuming breakfast (satiated state). Additionally, performance on the Food Stroop task and electrodermal activity (EDA) responses were measured to evaluate attentional interference and physiological arousal, respectively. Salivary samples were also collected to assess levels of alpha-amylase and cortisol.

**Results:**

Fasted participants exhibited a progressive decline in visual attention toward food stimuli compared to satiated individuals, reflecting a satiated state. This effect was independent of the palatability of the depicted food items and was not observed with stimuli representing non-food items. The Food Stroop task revealed no differences between fasting and satiated participants, indicating that the presence of food-related stimuli does not differentially impact attentional interference under varying hunger states. Moreover, no significant variations were observed in EDA responses across participant groups and stimulus types, suggesting that the modulation of visual attention to food cues by hunger is independent of physiological arousal. Interestingly, satiated subjects exhibited higher levels of salivary alpha-amylase, which was inversely related to their subjective hunger ratings. No differences in salivary cortisol levels were found between groups.

**Discussion:**

The findings indicate a novel influence of mild hunger on the processing of visual food cues, independent of physiological arousal. The decline in visual attention to food stimuli in fasted individuals suggests that satiety modulates visual processing. The lack of differences in attentional interference and physiological arousal between fasting and satiated states further supports the notion that visual attention to food cues is primarily driven by hunger-related mechanisms rather than stress. Additionally, the inverse relationship between salivary alpha-amylase levels and hunger ratings implies that alpha-amylase may serve as a marker of satiety rather than stress.

## Introduction

Food is a fundamental biological need for humans, and our visual system has evolved to detect and respond to visual food cues in our environment. Visual food stimuli are pervasive across various physical and online environments, including advertisements, shopping malls, and social media feeds. These stimuli not only shape our food preferences and consumption habits but also may foster addictive behaviors in susceptible individuals, particularly children (Smith et al., 2019; Arrona-Cardoza et al., 2023). Therefore, the omnipresent nature of these stimuli represents a significant concern as a potential environmental contributor to the current obesity epidemic.

Understanding how fasting affects visual attention to food cues can provide insights into the mechanisms of hunger-related changes in attention and their implications for eating behaviors. Motivational states finetune visual attention toward stimuli that align with specific drives (Hüttermann and Memmert, 2015; Vobrubová et al., 2023). Hunger modulates the processing of visual food stimuli, enhancing attentional selectivity toward images of food as opposed to non-food items (Channon and Hayward, 1990). Some studies further indicate attentional bias toward high energy foods (Castellanos et al., 2009; Doolan et al., 2014; Favieri et al., 2020; Phelan et al., 2011). However, others report no differential attentional bias between high energy and low energy stimuli (Graham et al., 2011) or even a heightened focus on low energy choices (Pedersen et al., 2021). Individual differences in responsiveness and reward sensitivity to food cues may underlie an increased risk of obesity in vulnerable individuals. Factors such as cognitive control, reward sensitivity, and affective states significantly affect how these cues are processed and responded to. This becomes particularly critical in environments abundant with high energy, highly palatable foods that are easily accessible and aggressively promoted. Consequently, numerous studies have explored the relationship between selective attention to food stimuli and body mass index (BMI). Obese individuals exhibit greater reward sensitivity toward food cues compared to lean individuals, potentially contributing to overeating and weight gain (Davis et al., 2011; Stice et al., 2013). This heightened sensitivity to food cues may result from changes in the brain’s reward processing and executive control systems in individuals with obesity (Vainik et al., 2013; Volkow et al., 2017).

Baseline data on physiological arousal in healthy, non-obese individuals, especially when comparing different states of hunger and satiety, are limited. Electrodermal activity (EDA) is a key measure of physiological arousal as it reflects sympathetic activity. EDA measures changes in the skin’s electrical conductance, influenced by the activity of sympathetically controlled eccrine sweat glands (Boucsein et al., 2012). Therefore, EDA can be employed as a highly sensitive and immediate indicator of emotional and autonomic responses. Some studies have shown differences in EDA responses to various food categories, such as high versus low energy foods (Pallavicini et al., 2016). Additionally, EDA has been observed to differ between food images with negative, positive or neutral valence, with heightened responses to aversive stimuli (Danner et al., 2014; Kuoppa, 2016; Verastegui-Tena et al., 2017). Other studies found no significant differences in EDA responses across food categories, nor a correlation between EDA responses and food reward or intake (Pedersen et al., 2021).

These findings highlight the need for further investigation into how physiological arousal, as measured by EDA, varies with different types of food cues and under different states of hunger and satiety in healthy-weight individuals.

The use of salivary biomarkers has been increasingly prominent in various research areas, including psychophysiology. Salivary biomarkers including cortisol and alpha-amylase are linked to both physiological arousal and eating behavior. While salivary cortisol is a traditional marker of HPA axis-dependent stress response, salivary alpha-amylase was more recently established as a marker of sympathetic-adrenal-medulary axis activation (Ali and Nater, 2020). Salivary cortisol was also linked to stress-related eating and high energy food cravings (Epel et al., 2001; Greeno and Wing, 1994), while salivary alpha amylase levels were recently found to be inversely associated with hunger and modulated by obesity (Moreno-Padilla et al., 2020).

This study aims to examine the influence of moderate 12-h fasting on the attentional processing of images categorized by their potential as food reward cues (non-food items, low energy food images, high energy food images), in healthy, non-obese participants. Our findings revealed that fasting leads to a reduced attention to food images over time, without affecting attention to non-food images, performance on the Food Stroop task, or electrodermal activity. To our knowledge, our study is the first to thoroughly explore the differences in physiological arousal to high and low energy food cues compared to non-food cues, while also distinguishing between hunger and satiety states in normal-weight, healthy individuals. Subsequently, it can be used to help inform studies on excessive weight individuals and food cues reactivity.

## Materials and methods

### Study participants

Participants were recruited through advertisements and posters displayed at the Faculty of Science, Charles University, Prague. The inclusion criteria required participants to be aged between 18 and 55 years, have a BMI between 18.5 and 25, be fluent in Czech at a native speaker level, and be capable of filling out questionnaires and performing computerized tasks. Exclusion criteria included pregnancy, severe psychiatric, neurological, or somatic diseases, current use of psychoactive medication, and any uncorrected vision impairments, including color vision deficiencies. Prior to the experimental session, volunteers completed a screening questionnaire to confirm their eligibility, from which their BMI was calculated based on self-reported height and weight. Out of 50 initially eligible participants, four were excluded due to incomplete or low quality eye-tracking or physiological data, or non-compliance with experimental protocols. The remaining 46 participants consisted of 26 women and 20 men, with a mean age of 28 ± 8 years and a normal BMI range (mean = 21 ± 1.7). Participants were randomly assigned to either the fasted group (*n* = 24), who were instructed not to eat or drink energy-containing beverages for 12 h prior to their session, or the satiated group (*n* = 22), who consumed their typical breakfast within two hours before their session. Each session lasted between 45 and 60 min and was conducted from 8 to 10 a.m. in a quiet, windowless room. The study adhered to the ethical standards of the Helsinki Declaration and was approved by the Institutional Review Board at Charles University (approval no. 2022/21). All participants were thoroughly informed about the experiment’s procedures and provided written informed consent before participating (see Supplementary materials).

### Study design

**Figure 1:**
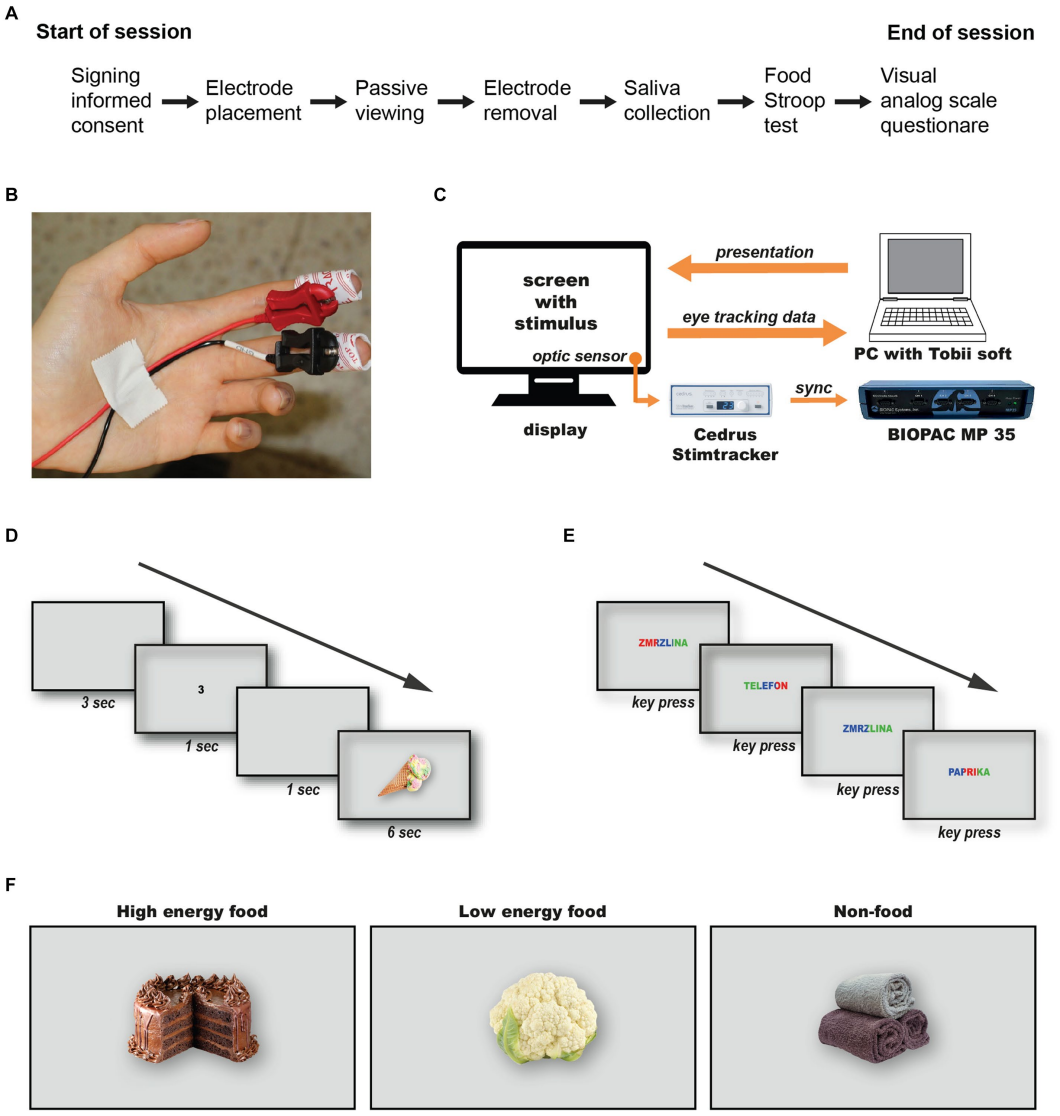
Data Acquisition and Experimental Design. The workflow for the experimental session **(A)**. Attaching disposable electrodes for electrodermal activity monitoring **(B)**. The configuration and synchronization method between the Tobii eye-tracking system and the Biopac data acquisition unit **(C)**. The timeline of stimulus presentation during the passive viewing test **(D)**, and the Food Stroop test **(E)**.

The experimental session workflow is depicted in Figure 1A. After providing informed consent, participants were asked to rinse their mouths (not swallow) with room temperature water that was prepared. Then participants were seated 60 cm from a computer screen and fitted with disposable BIOPAC EL507 electrodes on the fingertips of their non-dominant hand (Figure 1B). Electrodermal activity (EDA) was recorded using a BIOPAC MP35 data acquisition unit and Biopac Student Lab 4.1.3 software at a 200 Hz sampling rate. Eye-tracking data were captured using Tobii Studio software v3.4.8 on a color-calibrated 27” BenQ PD2700U IPS LCD monitor, with a Tobii X2-60 eye-tracker mounted on the monitor’s lower frame. Calibration was performed using a standard 9 point procedure as per the manufacturer’s instructions. Precise synchronization of electrophysiological and eye-tracking data was achieved using a Cedrus Stimtracker ST-100 with a light sensor attached to the screen’s bottom right corner (Figure 1C). In the passive viewing test, stimuli were presented against a consistent neutral background. Each stimulus belonged to one of three categories: high energy foods, low energy foods, or non-food items, with 30 stimuli per category based on caloric tables from the energys.info website (see Supplementary materials for stimulus details). All stimuli were matched for size, resolution, and depth of field, and were selected for their cultural relevance to the participant pool.

The session started with an instruction slide, followed by a 5 min relaxation period with a black screen. This was succeeded by a sequence comprising a 3 s grey screen, a random number (1, 2 or 3) displayed for 1 s, another 1 s grey screen, and finally the target stimulus shown for 6 s (Figure 1D). This sequence design allows ample time to capture stimulus-specific electrodermal reactions (Boucsein et al., 2012). The order of stimuli and numbers was pseudo-randomized for each participant. Following the passive viewing test, electrodes were removed, and saliva samples were collected using the Salimetrics Passive Drool Method. Samples were analyzed using the Expanded Range High Sensitivity Salivary Cortisol Enzyme Immunoassay kit (Salimetrics Pro #1-3002) and the Alpha-Amylase Saliva ELISA kit (BioVendor R&D RTC020R), with at least 1 ml of fluid collected and frozen at −20°C within 4 h. However, due to institutional COVID-19 restrictions, saliva collection was not permitted in the first two weeks, reducing the number of participants providing samples (*n* = 32). The session concluded with a Food Stroop test, custom-translated in Czech to include 36 words divided into categories of high energy foods, low energy foods, and neutral non-food objects (see Supplementary materials for both Czech and English versions). Each word was displayed once in each of three colours (blue, green, red) in a random sequence. Following a brief practice session using six hydrographic terms (e.g., “river,” “ocean,” etc.), participants identified the color of each word displayed on the screen by pressing a dedicated key on the keyboard (Figure 1E). After the test, participants completed a visual analogue scale questionnaire without numerical values adapted from Flint et al. (2000) to assess current hunger and satiety levels. Since the word “slaný” in the Czech language reflects both a salty and savory character of a meal, we combined questions on salty and savory preferences into one. Participants were compensated with a 100 CZK voucher for their participation, approximately lasting 45 to 60 min.

### Analysis of salivary samples

The saliva samples were collected and stored at −20°C until analysis. The day before analysis, samples were transferred to a refrigerator at 4°C. One hour before analysis, the samples and test kits were brought to room temperature. Concentrations of salivary markers were measured using the direct enzyme-linked immunosorbent assay (ELISA) method. The Expanded Range High Sensitivity Salivary Cortisol Enzyme Immunoassay kit (Salimetrics Assay #1-3002) and the Alpha-Amylase Saliva ELISA kit (BioVendor R&D, SKU: RTC020R) were used to analyse salivary cortisol and alpha-amylase, respectively, following the manufacturer-provided protocols. Data were analyzed using MyAssays software.^1^

### Analysis of electrodermal activity responses

For the analysis of electrodermal activity, we utilized AcqKnowledge 5.0.2 software (Biopac Systems, Inc., Goleta, CA, USA). Initially, the data were resampled at 50 Hz and subsequently filtered using a low-pass filter with a cutoff at 1 Hz to eliminate high-frequency noise. Following the protocol of Braithwaite et al. (2013), a threshold of 0.01 μS was employed to detect specific electrodermal responses. The data were then manually cleansed of artifacts, and several metrics of electrodermal responses were analyzed, including skin conductance level (SCL), skin conductance response (SCR) latency, SCR amplitude, and SCR rise time. Specific electrodermal responses (SCRs) were defined as those occurring within 1–6 s after stimulus presentation.

### Visual attention analysis

Tobii Studio software version 3.4.8 was utilized to analyze the eye-tracking and Food Stroop test data. For eye-tracking, total fixation durations for each stimulus and participant were exported. The reaction times for the Food Stroop test were calculated for each participant, defined as the time in seconds from the presentation of a word on the screen to the participant’s key press response.

### Statistical data analysis

All data were analyzed using GraphPad Prism version 8.0 for Windows (GraphPad Software, San Diego, California, USA). Statistical tests included unpaired t-tests, two-way ANOVAs, and either Sidak’s or Tukey’s post-hoc multiple comparison tests, as specified in the Results section. The significance levels for post-hoc tests are denoted in the graphs with asterisks (*p < 0.05, **p < 0.01, ***p < 0.0001); non-significance is indicated by ‘ns’. Pearson’s correlation coefficient (*r*), including a 95% confidence interval, determination coefficient (*r*^2^), and significance level, was calculated for correlated data. Unless otherwise specified, data are presented as mean ± SEM. The Visual Analog Scale (VAS) questionnaire responses were evaluated using unpaired t-tests to compare the means between the fasted and satiated groups. Significance levels were adjusted for multiple comparisons using the Benjamini, Krieger, and Yekutieli procedure, controlling the false discovery rate (FDR) at 5%.

## Results

### Attentional responses to passive viewing of visual stimuli

**Figure 2:**
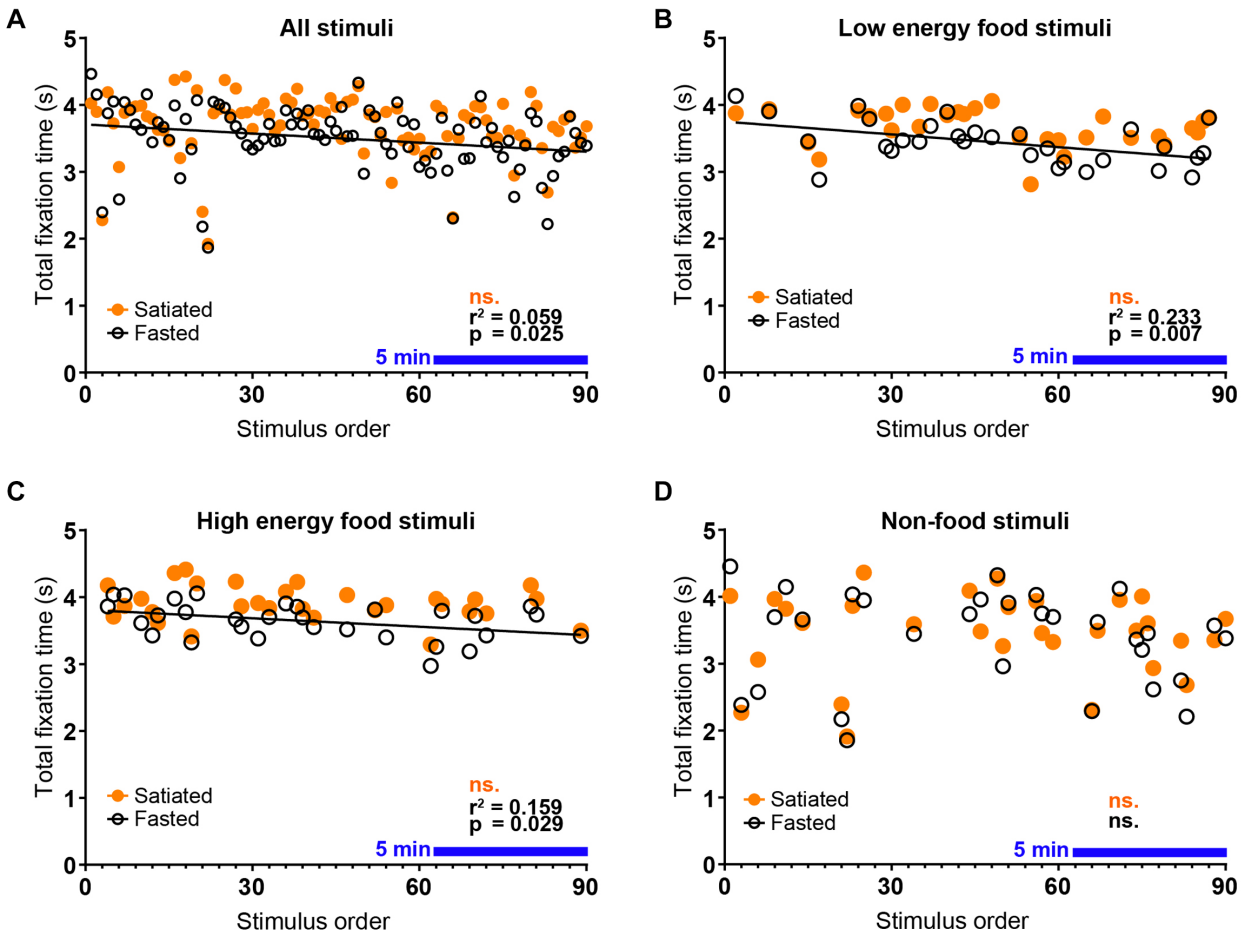
Decay of Visual Attention to Food Stimuli in Fasted Individuals. Eye-tracking data demonstrate a rapid decline in visual attention to food stimuli among fasted participants. A negative correlation was observed between the total fixation time per stimulus and the order of stimulus presentation within the passive viewing experimental block, indicating that attention decreased over time in the fasted group but not in the satiated group **(A)**. This pattern was consistent across food categories, with significant correlations found for both low energy **(B)** and high energy **(C)** food stimuli in the fasted group. In contrast, no correlation was detected between stimulus order and total fixation time for non-food stimuli **(D)**, confirming that the observed decline in visual attention is specific to food stimuli and dependent on satiety state. The blue line indicates a continuous period of 5 minutes.

The experimental results demonstrate a progressive decline in visual attention throughout the session, particularly among fasting participants. This trend, shown by the decreasing total duration of visual fixations with successive stimulus presentations (Figure 2A), was exclusive to the fasted group. Detailed analysis confirmed that this decline was specific to food stimuli, regardless of their energy content (Figures 2B–D). The reduction in visual attention was statistically significant in the fasted group [Pearson’s *r*(88) = −0.2426, *r*^2^ = 0.05883, *p* = 0.0213], whereas no significant changes were detected in the satiated group (*p* = 0.2457). Both low energy [Pearson’s *r*(28) = −0.4831, *r*^2^ = 0.2334, *p* = 0.0068] and high energy [Pearson’s *r*(28) = −0.3988, *r*^2^ = 0.1590, *p* = 0.0291] food stimuli exhibited notable declines in attention within the fasted group. In contrast, visual attention changes for both low energy (*p* = 0.1782) and high energy (*p* = 0.2721) stimuli were not significant in the satiated group, nor were the changes for non-food stimuli in either group (Fasted: *p* = 0.6724, Satiated: *p* = 0.8961).

**Figure 3:**
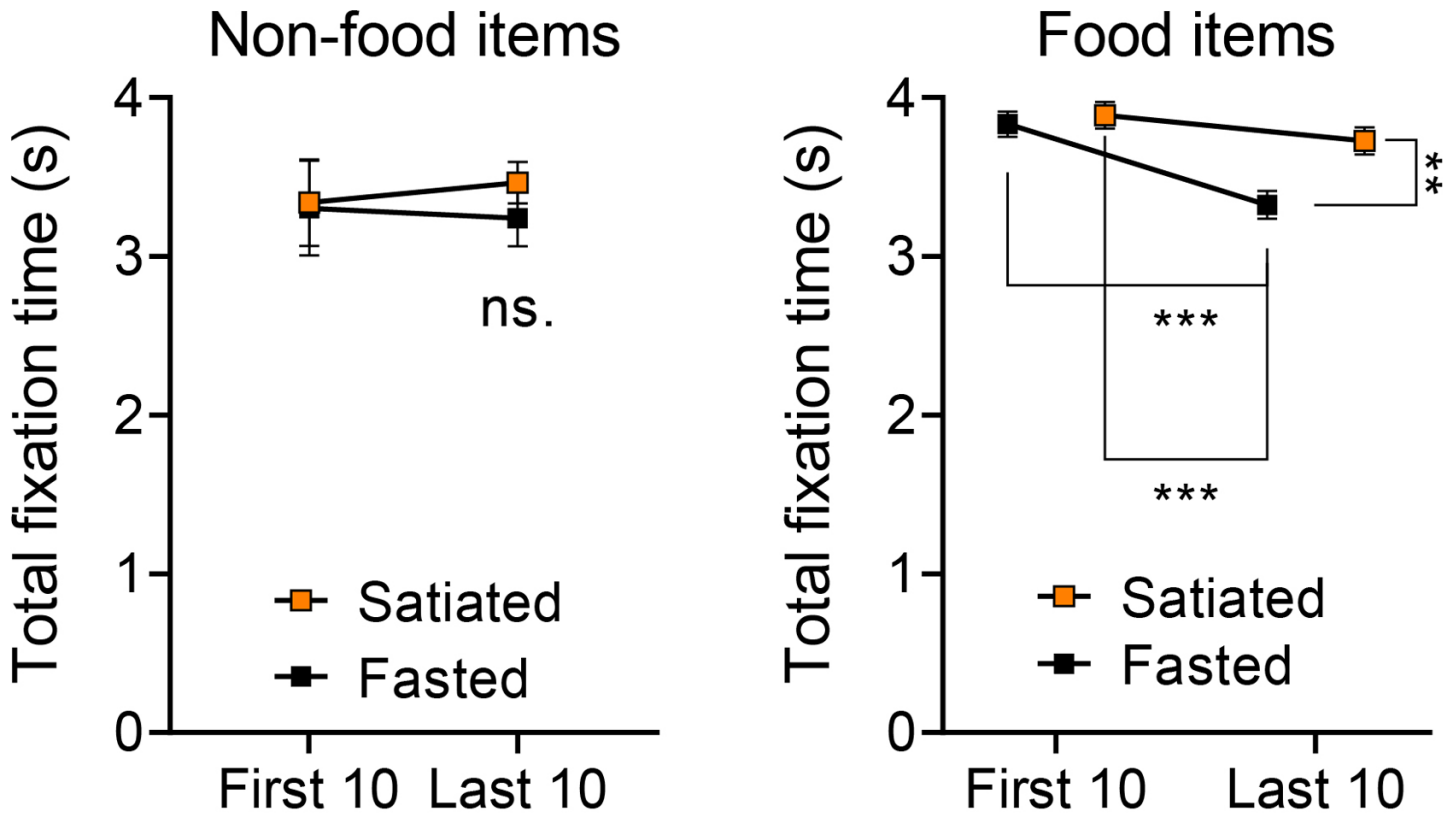
Visual Attention Across First 10 and Last 10 Stimuli in Non-Food and Food Categories. To confirm the trends identified in the correlation analysis, we further examined differences in visual attention across the first 10 and last 10 stimuli for both non-food and food categories. In the case of non-food items, no differences were observed between the first 10 and last 10 stimuli or across participant groups (left). Conversely, a two-way ANOVA followed by Tukey’s post-hoc test indicated that, for food items (encompassing both low and high energy categories), the last 10 items presented to the fasted group garnered significantly reduced total fixation times compared to all other stimulus groups (right).

Further comparisons of the total visual fixation durations for the first and last ten stimuli categorized by Non-food vs. Food items (Figure 3) revealed no significant differences in the Non-food category via a two-way ANOVA. However, significant findings emerged in the Food category, including a main effect of stimulus order [*F*(1, 36) = 15.83, *p* = 0.0003], a main effect of satiety state [*F*(1, 36) = 7.478, *p* = 0.0096], and an interaction effect [*F*(1, 36) = 4.262, *p* = 0.0462]. Tukey’s post-hoc test indicated significant differences between the first and last ten food stimuli for the fasted group (*p* = 0.0007), between the first ten stimuli for the satiated group and the last ten for the fasted group (*p* = 0.0002), and between the last ten stimuli for both satiated and fasted groups (*p* = 0.0088).

### Food Stroop test

**Figure 4:**
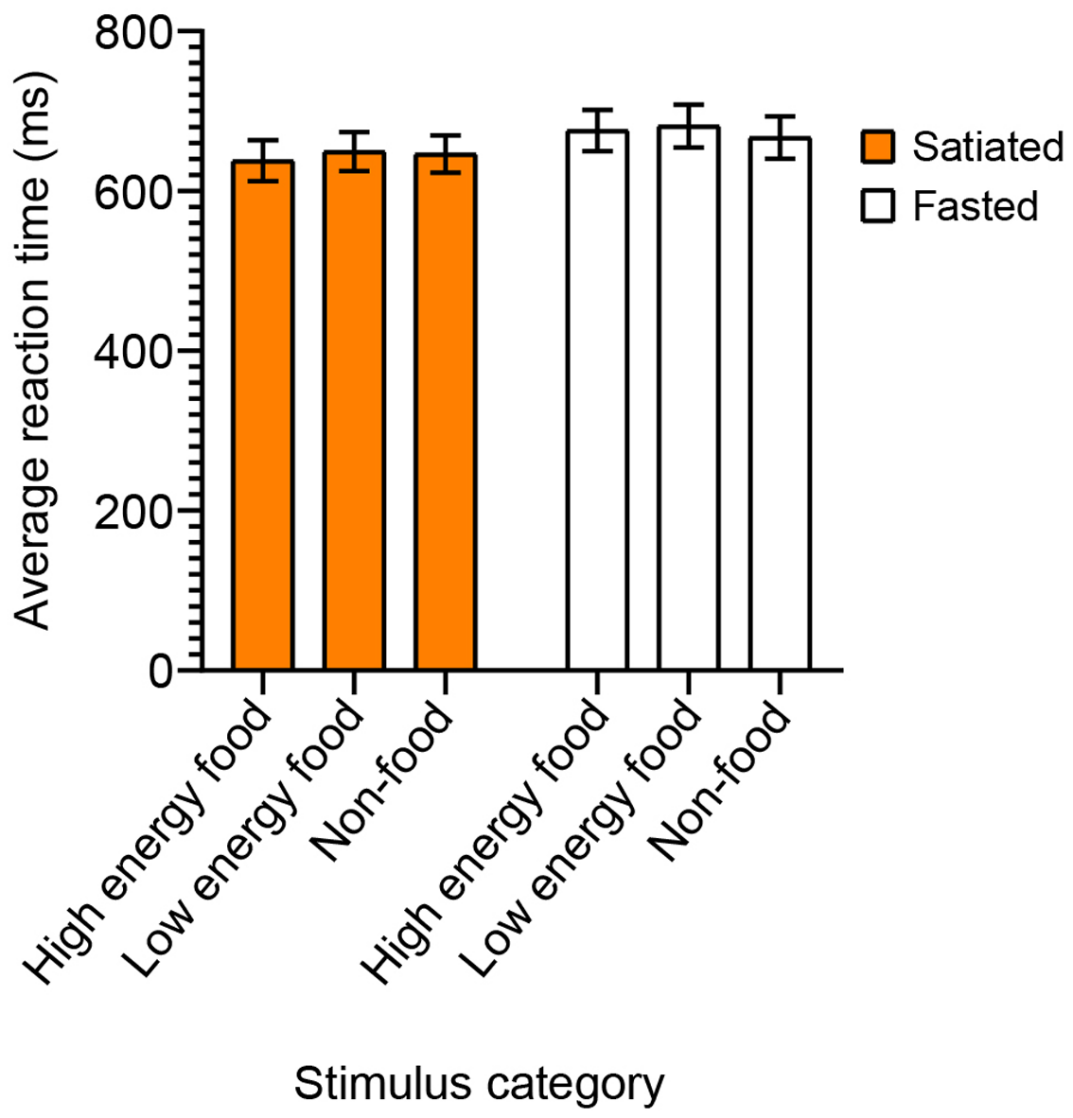
Reaction Times in the Food Stroop Test by Stimulus Category. To evaluate the impact of satiety state on attentional interference from food cues, we conducted the Food Stroop test. Satiated participants are represented by a solid orange box, while fasting participants by an empty box. No significant differences in reaction times were found between the satiated and fasting groups across any of the stimulus categories.

In the Food Stroop test, a two-way ANOVA with Sidak’s post-hoc test for multiple comparisons was conducted to evaluate the mean reaction times based on satiety status and stimulus type (Figure 4). The analysis did not reveal any statistically significant effects of satiety [*F*(1, 132) = 2.093, *p* = 0.1503] or stimulus category [*F*(2, 132) = 0.07552, *p* = 0.9273].

### Electrodermal activity

**Figure 5:**
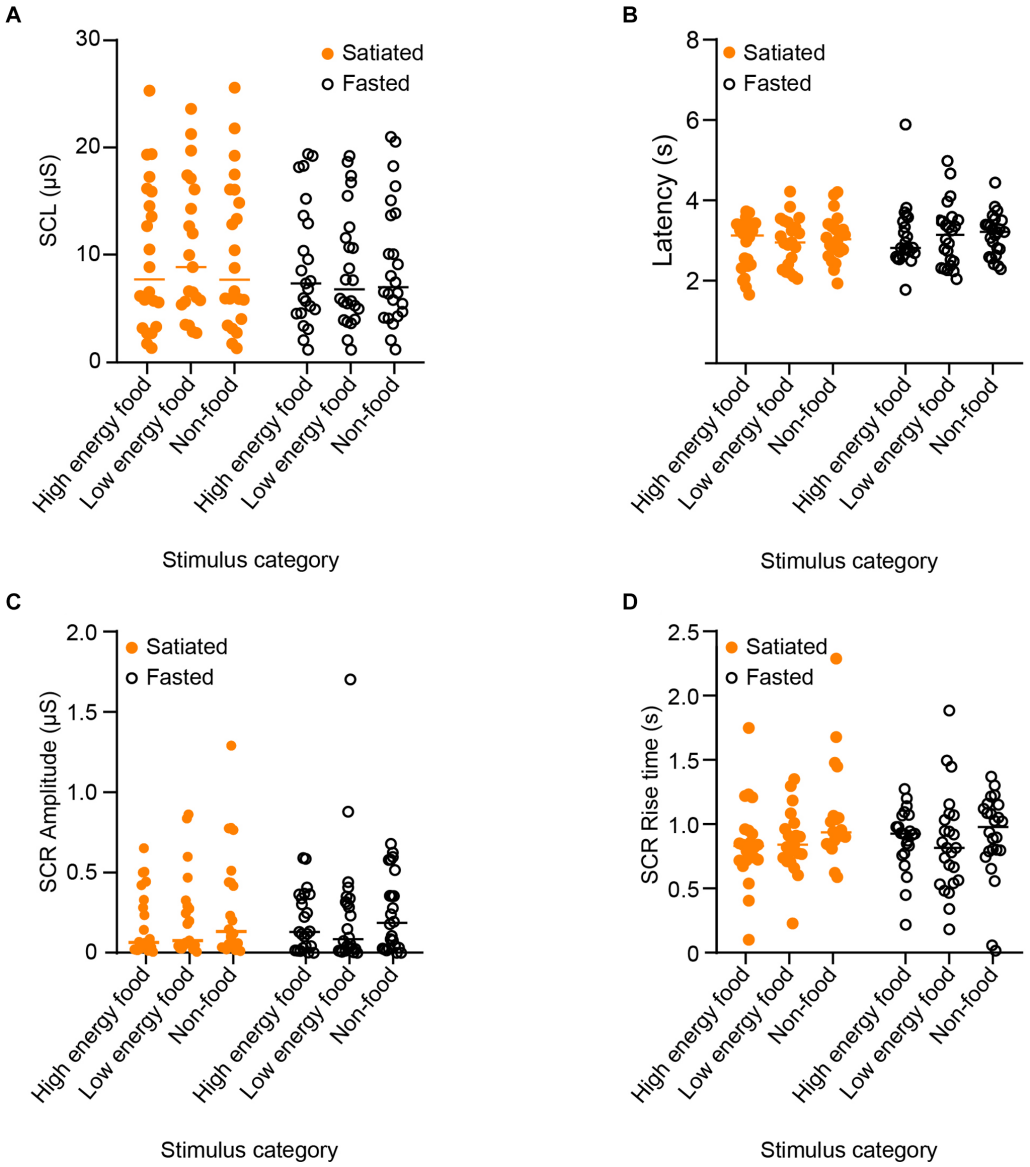
Parameters of Electrodermal Activity in Response to Stimuli by Category. To evaluate physiological arousal and its potential relationship with visual attention, we measured electrodermal activity responses across different stimulus categories. The analyzed parameters include skin conductance level (SCL) in **(A)**, skin conductance response (SCR) latency **(B)**, SCR amplitude **(C)**, and SCR rise time **(D)**. Satiated participants are represented by solid orange points, and fasting participants by empty black circles. No significant differences were observed across participant groups or stimulus categories.

The results for electrodermal activity are presented in Figure 5. We utilized a two-way ANOVA followed by Sidak’s post-hoc test for multiple comparisons to analyse the impact of stimulus type and satiety on skin conductance level (SCL). Neither satiety (*F* (1, 130) = 0.007535, *p* = 0.9925) nor stimulus category [*F*(2, 130) = 1.180, *p* = 0.2793] had a significant effect on SCL. The effects on latency were also evaluated. No significant impacts were observed for satiety [*F*(1, 130) = 2.233, *p* = 0.1375] or stimulus category [*F*(2, 130) = 0.3957, *p* = 0.6740], indicating no statistical significance in either case.

In addition, we assessed the influence of satiety and stimulus type on skin conductance response (SCR) amplitude. Results showed that neither satiety level [*F*(1, 130) = 0.0001489, *p* = 0.9903] nor stimulus category [*F*(2, 130) = 1.0703, *p* = 0.3449] significantly affected SCR amplitude. Finally, SCR rise time was not significantly influenced by either satiety [*F*(1, 130) = 0.3124, *p* = 0.5772] or stimulus category [*F*(2, 130) = 2.008, *p* = 0.1384].

### Subjective evaluation of hunger, satiety, and food cravings

Subjective assessments of hunger, satiety, and food cravings were conducted using the Visual Analog Scale, as detailed in Table 1. Statistical analysis was performed with unpaired t-tests and adjusted for multiple comparisons using the Benjamini, Krieger, and Yekutieli method (FDR = 5%). Significant differences emerged, with fasted individuals reporting higher levels of hunger (*p* = 0.0101, *q* = 0.00796). In contrast, satiated participants reported significantly greater feelings of satisfaction (*p* < 0.0001, *q* = 0.00011) and fullness (*p* = 0.0021, *q* = 0.00338). There were also marked differences in the cravings for sweet (*p* = 0.0087, *q* = 0.00796) and savory (*p* = 0.0289, *q* = 0.0182) foods, with stronger proclivities in the fasted group. No significant differences were found in the desire for spicy (*p* = 0.9933, *q* = 0.3911) or fatty foods (*p* = 0.1254, *q* = 0.0564) across the groups.

**Table 1:** Self-assessment scores based on the Visual Analog Scale questionnaire, comparing responses from the fasted and satiated participants.

Question	Mean of hungry	Mean of satiated	Difference	*p* value	*Q* value (FDR 5%)
Q1: How hungry do you feel?	5.588	3.496	2.092	0.010111	**0.007962**
Q2: How satisfied do you feel?	2.267	5.314	−3.047	0.000035	**0.00011**
Q3: How full do you feel?	2.188	4.542	−2.354	0.002144	**0.003376**
Q4: How much do you think you can eat?	7.221	5.95	1.271	0.064959	0.034104
Q5: Would you like to eat something sweet?	6.9	4.492	2.408	0.008681	**0.007962**
Q6: Would you like to eat something salty/savory?	7.856	6.163	1.694	0.028886	**0.018198**
Q7: Would you like to eat something spicy?	4.204	4.213	−0.008333	0.993278	0.391103
Q8: Would you like to eat something fatty?	5.292	3.842	1.45	0.125374	0.056418

Displayed are questions in the English language, the mean values, the differences between states, and statistical significance determined through unpaired t-tests (*p* values) and adjusted for multiple comparisons (Q values) using the procedure or Benjamini, Krieger, and Yekutieli with a 5% FDR threshold. Bold Q values indicate statistical significance.

### Salivary levels of cortisol and alpha-amylase

**Figure 6:**
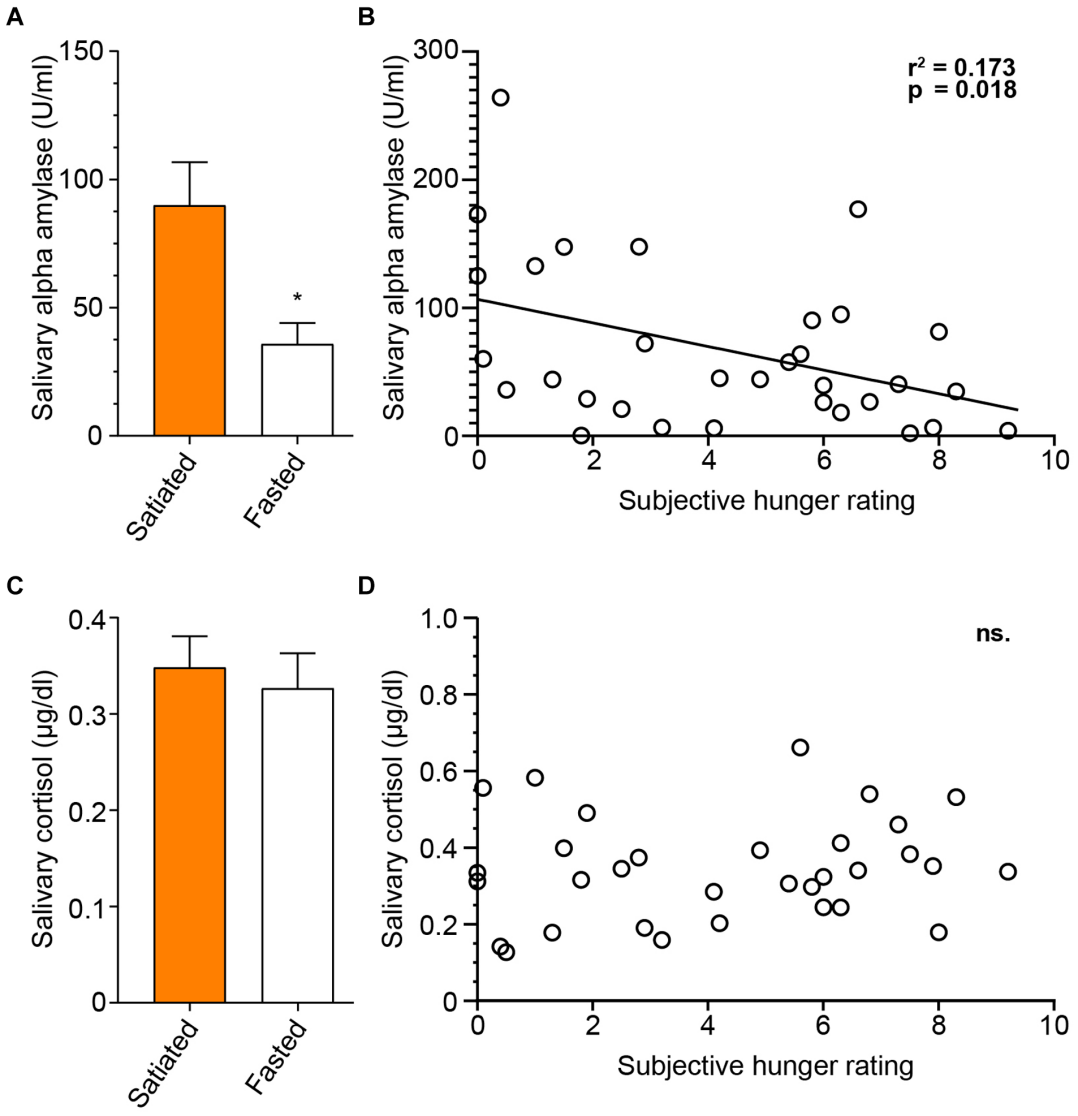
Salivary Alpha-amylase, but not Cortisol is Associated with Hunger State. To assess salivary markers of satiety and stress response, we analyzed saliva samples collected at the conclusion of the experimental session, measuring levels of salivary alpha-amylase and cortisol. Concentrations of salivary alpha-amylase differed significantly between participant groups **(A)**, and alpha-amylase levels were correlated with self-evaluated hunger **(B)**. Salivary cortisol concentrations did not differ between groups **(C)**, and no association between post-session cortisol levels and self-evaluated hunger was found **(D)**. Satiated participants are represented by a full orange box, and fasting participants by an empty box.

Salivary markers were measured after the session to compare fasted and satiated participants (Figure 6). An unpaired t-test showed that cortisol levels did not significantly differ between the groups [*t*(30) = 0.4399, *p* = 0.6632]. In contrast, alpha-amylase levels were significantly higher in the satiated group than in the fasted group [*t*(30) = 2.640, *p* = 0.0130].The relationship between subjective hunger and salivary markers was also assessed, using the Visual Analog Scale self-assessment for hunger (Q1: ‘How hungry do you feel?’). There was no correlation between cortisol levels and subjective hunger (*p* = 0.4301). However, a significant negative correlation was found between alpha-amylase levels and hunger ratings [Pearson’s *r*(30) = − 0.4155, *r*^2^ = 0.1727, *p* = 0.0180].

## Discussion

In this study, we examined the influence of the post-prandial state on visual attention toward food and non-food stimuli in non-obese individuals. Participants either fasted for 12 h or consumed a meal of their choice within two hours before the experimental session. Given the variability in hunger induction protocols across different studies, we needed to validate that our 12h fast effectively elicited mild hunger in our participants. To achieve this, we employed the Visual Analog Scale (VAS). The VAS self-assessment questionnaire (Table 1) confirmed that fasting participants reported increased feelings of hunger and cravings for savory and sweet foods, as well as decreased sensations of satiety and fullness.

Our findings reveal that fasting individuals exhibit a distinct temporal pattern of visual attention to food stimuli, in contrast to those who had recently eaten. Specifically, we observed a progressive decline in visual attention among hungry participants, marked by increasingly shorter total fixation durations on food-related stimuli (Figures 2, 3). This decline was unique to food cues, regardless of the depicted food’s energy content, from high energy foods like chocolate cake to lower-energy foods such as raw cauliflower (Figure 1F). Conversely, attention toward non-food-related stimuli, e.g., a picture of three rolled up towels, remained consistent over time for both groups.

Previous studies have explored the impact of hunger on attentional selectivity to food stimuli, with varying outcomes. Recently, Weng et al. (2019) discovered that hunger influences binocular rivalry when viewing food-related images, suggesting that hunger exerts top-down modulation on visual processing at a subconscious level. Interestingly Castellanos et al. (2009) investigated attention patterns to food stimuli among obese and normal-weight women under both fed and fasted conditions (minimum of 8 h) using pairs of stimuli (food and non-food) that were matched in color, complexity, and brightness. Their findings revealed that normal-weight women demonstrated longer visual fixations on food stimuli, rather than the paired non-food stimuli, when hungry compared to their fed state. In contrast, obese women showed consistently elevated visual attention to food stimuli regardless of their satiety state. Unlike Castellanos et al., our study did not detect increased attention to food images among hungry participants. However, in contrast to their findings, we observed a decay of attention over time. The seeming discrepancy is likely attributable to differences in methodological approach: while our study utilized a sequence of individual images and explored a temporal aspect of visual attention, Castellanos et al. investigated attentional bias using paired stimuli. This attentional bias paradigm enables the comparison of visual attention between two different stimuli presented simultaneously, while the sequential presentation of stimuli offers the advantage of measuring stimulus-specific physiological arousal. Therefore, instead of contradicting each other, it seems that each study sheds light on different aspects of how hunger modulates visual attention to food cues.

Neal et al. (2023) found no evidence that hunger increases attentional capture by food cues using the Emotional Blink of Attention (EBA) task. However, in their study, participants fasted for the minimum of 6 h in contrast to 12 h in our study. Thus, hunger-related attentional biases may not be substantial over shorter intensities and durations. This further emphasizes the need to validate fasting protocol efficacy in eliciting mild hunger using self-evaluation measures like the Visual Analog Scale (VAS). A recent study by Ciorli et al. (2024) examined how hunger and energy content influence visual awareness of food stimuli using breaking Continuous Flash Suppression (bCFS) and Binocular Rivalry (BR) paradigms. They found that high energy foods accessed visual awareness faster, particularly in satiated participants, and dominated visual awareness longer, regardless of hunger state. These findings suggest that both energy content and hunger state significantly modulate the visual processing of food cues, supporting our observation that hunger and satiety influence attentional responses to food stimuli (Ciorli et al., 2024).

It is noteworthy that our study identified no attentional differences between images of high energy and low energy foods. While multiple studies support a focus on food-related cues over non-food cues (Doolan et al., 2014; Favieri et al., 2020; Nummenmaa et al., 2011), and a preference for high energy over low energy images is well-documented (Castellanos et al., 2009; Doolan et al., 2014; Favieri et al., 2020; Phelan et al., 2011), discrepancies remain. For instance, some research has shown no bias toward high energy stimuli (Graham et al., 2011) or even a preference for low energy stimuli (Pedersen et al., 2021). The absence of an expected attentional preference for high energy foods in our study could be attributed to two factors: firstly, our participants were healthy and non -obese; secondly, our experimental approach differed from other studies that typically assess attentional biases using pairs of images presented simultaneously, whereas we presented single images sequentially. Investigating whether individuals at risk of obesity and food addiction would exhibit a preference for high energy images in this paradigm warrants further research.

Several studies observed differential modulation of visual attention food cues by hunger in healthy individuals as opposed to individuals suffering from obesity and eating disorders. Nummenmaa et al. (2011) demonstrated that the human visual system is specialized in detecting food targets among non-food items, with a significant correlation between faster detection of food items and lower Body Mass Index (BMI). Their study found that individuals with lower BMI showed a stronger attentional bias toward food stimuli, suggesting that the attentional system is evolutionarily tuned to prioritize nutriments, which might vary based on an individual’s energy needs and body mass. Our study’s finding that hungry individuals exhibit attentional desensitization to visual food cues can be interpreted as a form of strategic disengagement, aligning with Nijs and Franken’s (2012) hypothesis of attentional biases in overweight and obese individuals. They described an approach –avoidance pattern where initial strong attention to high energy food stimuli is followed by deliberate disengagement, likely to manage the conflict between the immediate appeal of food and long-term goals like weight control. Similarly, in our study, hungry individuals may initially show heightened attention and arousal to food cues, but over time, exhibit reduced engagement as an adaptive mechanism to prevent overwhelming preoccupation with food. The modulation of visual attention by hunger is likely to have direct effect on food choices and eating behaviors. Dai et al. (2020) explored how perceptual salience impacts food choices, showing that individuals are more likely to choose healthier food options when they are made visually salient, even under high cognitive load or time pressure. This study demonstrates the significant role of visual attention in food-related decision-making, aligning with our findings that hunger and attentional mechanisms are critical in determining responses to food cues. These data suggest that visual salience could be a valuable tool in interventions aimed at promoting healthy eating behaviors. The Food Stroop task has been pivotal in assessing attentional bias toward food stimuli. Previous studies by Channon and Hayward (1990) and Lavy and van Den Hout (1993) demonstrated that hunger increases reaction times, suggesting a hunger-induced attentional bias. However, in our study, both fasted and satiated participants performed similarly in the Food Stroop task, indicating that moderate hunger does not significantly affect attentional interference from food-related stimuli in healthy, non-obese individuals (Figure 4). Furthermore, we observed no prolonged reaction times in hungry individuals nor differences across stimulus categories, which could be attributed to the mild nature of fasting in our study and the health profile of our non-obese participants. Nijs et al. (2010) investigated attentional biases to food-related words using a modified Stroop task and event-related potentials (ERPs) in obese and normal-weight individuals. They found that obese participants displayed a larger P200 amplitude to food-related words, indicating an enhanced automatic, preconscious attentional orientation toward food stimuli compared to neutral words, a pattern not observed in normal-weight participants. This enhanced automatic attention to food cues in obese individuals supports the idea that early stages of attentional processing are crucial in understanding eating behaviors, aligning with our findings that hunger modulates attentional and physiological responses to food cues in non-obese individuals, thereby highlighting the need to consider individual differences in attentional mechanisms related to food cues.

In our study, satiated participants showed elevated salivary alpha-amylase levels, inversely correlated with subjective hunger scores, but unchanged levels of salivary cortisol. While our findings associate mild hunger with reduced secretion of salivary alpha-amylase—a marker traditionally linked to physiological arousal and stress—we contend that these alpha-amylase variations are more directly related to hunger levels than to physiological arousal. This conclusion is supported by several observations: firstly, no changes in electrodermal activity were linked to hunger or type of stimulus; secondly, no correlation was found between hunger and cortisol levels; thirdly, there was a direct negative correlation between subjective hunger ratings and alpha-amylase levels; and fourthly, it seems implausible that the satiated group would exhibit higher levels of arousal than the hungry group and not vice versa. Our results are thus in accordance with Moreno-Padilla et al. (2020) who found significant negative correlation between hunger and salivary alpha-amylase after viewing food images. Thus, our data corroborate the finding that alpha-amylase is a marker of satiation, independent of physiological arousal and stress levels. A limitation of our study is that measuring subjective hunger after viewing food images makes it unclear whether the correlation between salivary alpha-amylase levels and hunger is a general phenomenon or specific to the context of viewing food images.

Taken together, our study reports a novel phenomenon of hunger-induced attentional desensitization to food cues. While multiple earlier studies reported attentional modulation to visual food cues by hunger specifically in obese populations, we found that the hunger-induced attentional desensitization to food cues is a robust phenomenon that extends beyond overweight and obese individuals to normal-weight. Collectively, these studies suggest the potential usefulness of studying hunger-induced attentional desensitization not only in normal weight, healthy individuals, but particularly in individuals with or at risk of obesity and food addiction. This paradigm may provide valuable insights into the underlying cognitive and physiological mechanisms of food –related attention and eating behaviors. Furthermore, understanding hunger-induced attentional desensitization could inform the development of targeted interventions aimed at managing attentional biases and reducing overeating in at-risk populations. Future research should explore the long-term implications of this desensitization on eating patterns and weight management, as well as potential differences in neural correlates between normal-weight and overweight individuals and potentially also in individuals suffering from eating disorders characterized by periodic overeating, such as binge eating disorder and bulimia nervosa.

## Data Availability

The original contributions presented in the study are included in the article/Supplementary material, further inquiries can be directed to the corresponding author.

## Footnotes

^1^ myassays.com
