# Supplementary material for: Mild hunger elicits attentional desensitization to visual food cues in healthy, non-obese individuals
Source: Front Psychol. 2024 Sep 9;15:1441184. doi: 10.3389/fpsyg.2024.1441184 (PMC11417041; doi:10.3389/fpsyg.2024.1441184)
Supplement: Supplementary file 1 [file Data_Sheet_1.PDF]

SUPPLEMENTARY MATERIALS

Informovaný souhlas o účasti v experimentu

Přihlásili jste se k dobrovolné účasti ve vědeckém experimentu „Psychofyziologické koreláty vyvolané vizuálními potravinovými stimuly během hladu a sytosti“ jehož účelem je monitorování fyziologických odpovědí na obrazové podněty prezentované na monitoru počítače. V rámci experimentu Vám budou promítány obrázky a současně bude snímáno Vaše EKG a elektrodermální kožní odpověď a odebírány sliny před prvním blokem a také po skončení prvního bloku. Dále budou kamerou monitorovány Vaše oční pohyby.

Procedury snímání EKG a elektrodermální odpovědi nepředstavují zdravotní riziko, pokud netrpíte zvýšenou kožní dráždivostí např. z důvodu ekzému či poranění, která by Vám mohla způsobit obtíže při kontaktu kůže s nalepovací elektrodou. Tyto elektrody jsou zdravotně nezávadné a běžně se používají v klinické praxi. Veškerá v tomto experimentu použitá měřící zařízení jsou akreditována pro použití v humánních experimentech.

Než se rozhodnete, zda se chcete studie zúčastnit, je nezbytné, abyste byl(a) o studii dostatečně informován(a). Je povinností vyšetřujícího technika vyjasnit veškerá fakta v rámci podrobného osobního rozhovoru. Pokud budete mít jakékoliv další dotazy, neváhejte se nyní zeptat. O své účasti se rozhodujte bez spěchu, po důkladném uvážení. Vaše účast v této studii je čistě dobrovolná a máte právo ji kdykoliv přerušit bez udání důvodu. Experimentální postupy prováděné v rámci tohoto experimentu byly schváleny institucionální etickou komisí Přírodovědecké fakulty Univerzity Karlovy.

Prosíme, abyste podepsal(a) informovaný souhlas, pokud:

1. Jste plně porozuměl(a) povaze experimentu a procedurám, které jsou v něm zahrnuty
2. Souhlasíte se svou účastí na experimentu
3. Znáte svá práva coby účastníka experimentu

Podpisem níže stvrzuji svou dobrovolnou účast na vědeckém experimentu „Psychofyziologické koreláty vyvolané vizuálními potravinovými stimuly během hladu a sytosti“:

Jméno a příjmení účastníka experimentu	Podpis účastníka experimentu	Datum (den, měsíc, rok)
______________________________	_____________________	__________________

Informed consent to participate in the experiment

You have volunteered to participate in the scientific experiment "Psychophysiological correlates of visual food stimuli during hunger and satiety" whose purpose is to monitor physiological responses to visual stimuli presented on a computer monitor. As part of the experiment, images will be projected to you, and at the same time your ECG and electrodermal skin response will be recorded, and saliva will be collected before the first block and also after the end of the first block. Furthermore, your eye movements will be monitored by the camera.

The ECG and electrodermal response procedures do not pose a health risk if you do not suffer from increased skin irritation, e.g. due to eczema or an injury, which could cause you difficulties when your skin comes into contact with the adhesive electrode. These electrodes are harmless to health and are commonly used in clinical practice. All measuring devices used in this experiment are accredited for use in human experiments.

Before you decide whether you want to participate in the study, it is essential that you are sufficiently informed about the study. It is the responsibility of the investigating technician to clarify all the facts in a detailed personal interview. If you have any other questions, don't hesitate to ask now. Make decisions about your participation without haste, after careful consideration. Your participation in this study is purely voluntary and you have the right to withdraw at any time without giving any reason. The experimental procedures carried out as part of this experiment were approved by the institutional ethics committee of the Faculty of Science, Charles University.

Please sign the informed consent if:

1. You fully understand the nature of the experiment and the procedures involved

2. You agree to your participation in the experiment

3. You know your rights as a participant in the experiment

By signing below, I confirm my voluntary participation in the scientific experiment "Psychophysiological correlates of visual food stimuli during hunger and satiety":

Name and surname of the experiment participant	Signature of the experiment participant	Date (day, month, year)
________________________________	______________________	___________________

DOTAZNÍK E1007
Subjekt:

Jak hladový/á se cítíte?

Vůbec nejsem hladový/á	______________________	Nikdy jsem nebyl/a víc hladový/á

Jak nasycený/á se cítíte?

Už nesním ani kousek	______________________	Cítím se úplně prázdný/á

Jak plný/á se cítíte?

Cítím se úplně plný/á	______________________	Vůbec se necítím plný/á

Kolik si myslíte, že můžete sníst?

Vůbec nic	______________________	Hodně

Dal/a byste si k jídlu něco sladkého?

Ano, velice rád/a	______________________	Vůbec ne

Dal/a byste si k jídlu něco slaného?

Ano, velice rád/a	______________________	Vůbec ne

Dal/a byste si k jídlu něco pikantního?

Ano, velice rád/a	______________________	Vůbec ne

Dal/a byste si k jídlu něco tučného?

Ano, velice rád/a	______________________	Vůbec ne

QUESTIONNAIRE E1007
Subject:

How hungry do you feel?

I am not hungry at all ______________________________ I have never been more hungry

How satisfied do you feel?

I cannot eat another bite ______________________________ I am completely empty

How full do you feel?

Totally full ______________________________ Not at all full

How much do you think you can eat?

Nothing at all ______________________________ A lot

Would you like to eat something sweet?

Yes, very much ______________________________ No, not at all

Would you like to eat something salty?

Yes, very much ______________________________ No, not at all

Would you like to eat something spicy?

Yes, very much ______________________________ No, not at all

Would you like to eat something fatty?

Yes, very much ______________________________ No, not at all

High-calorie food stimuli

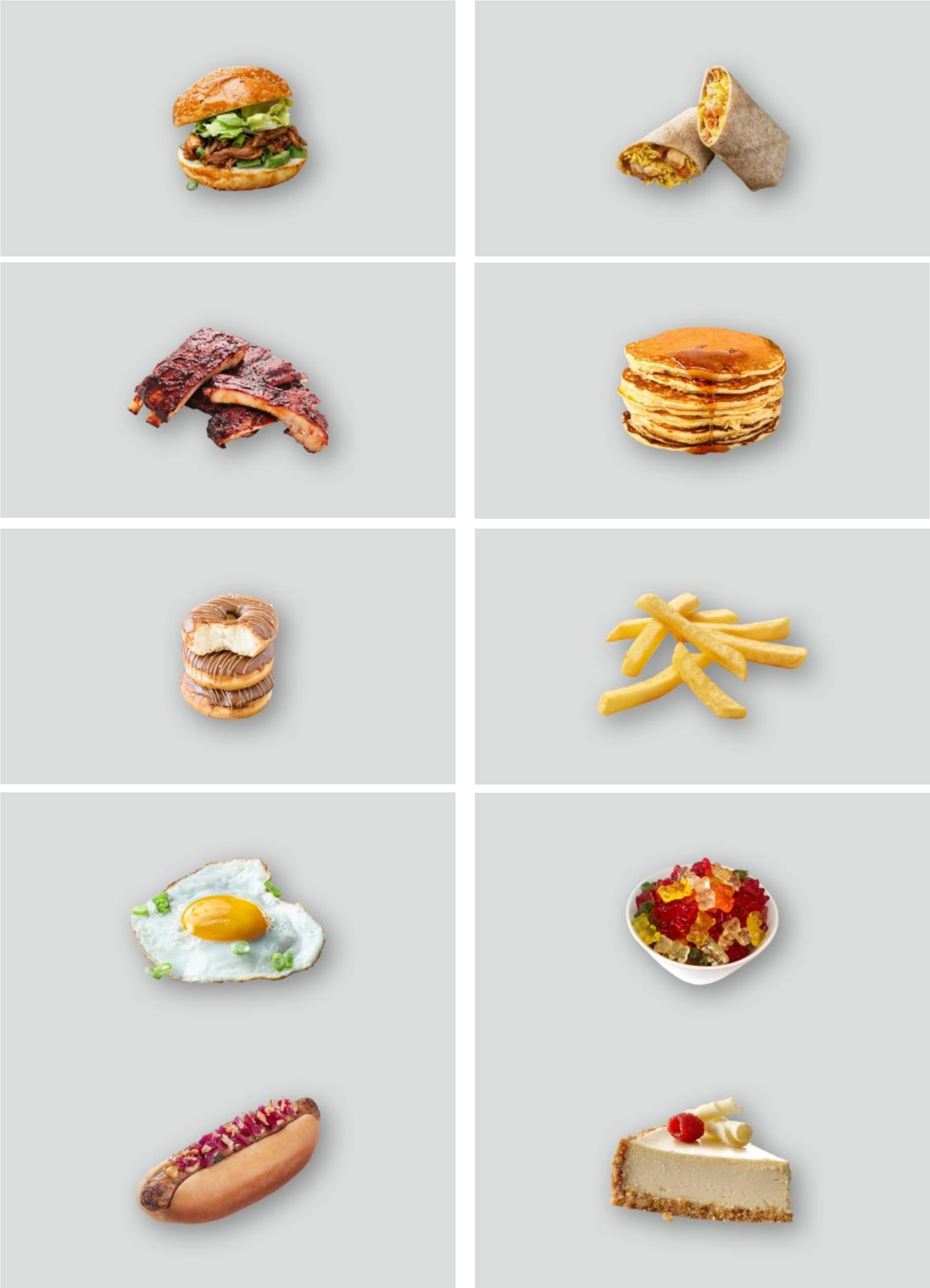

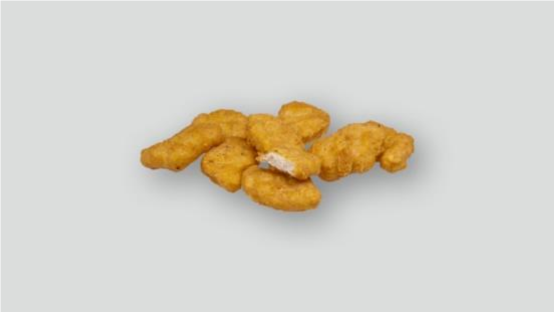
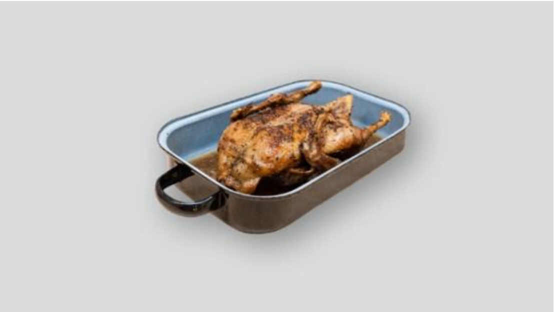
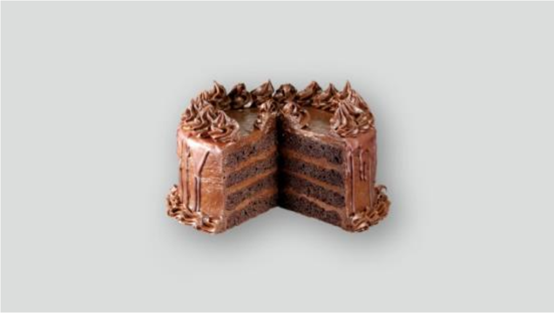
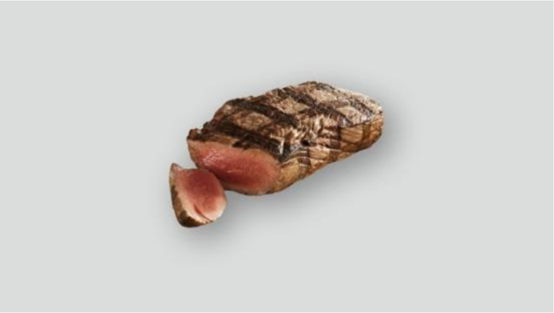
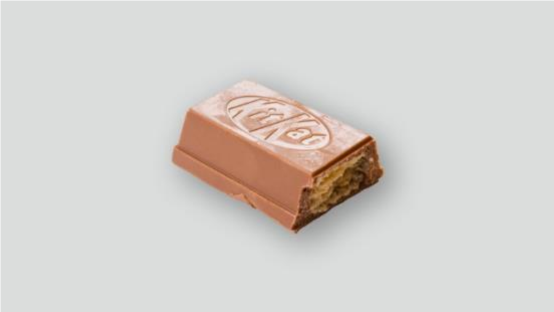
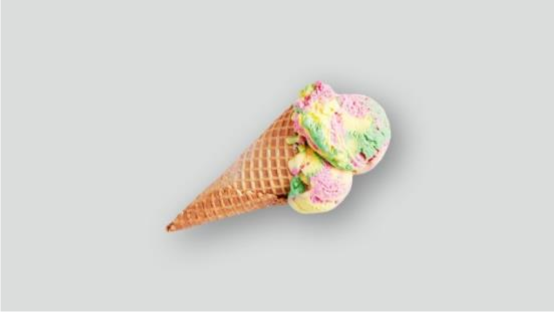
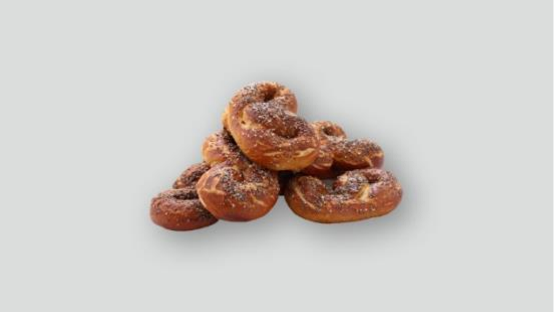
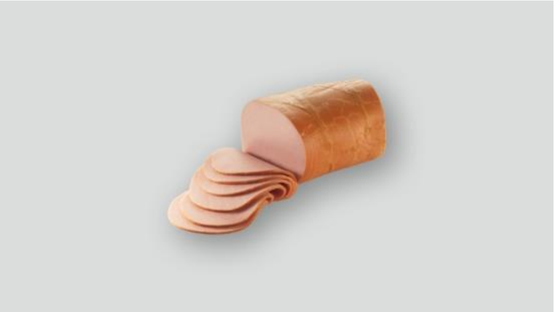
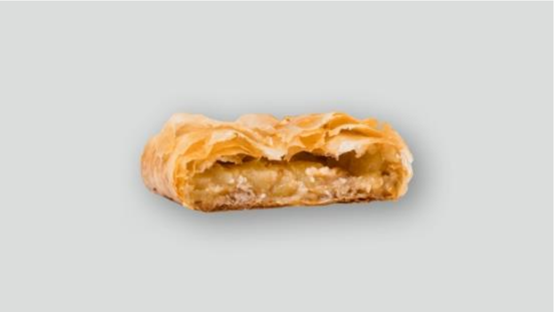
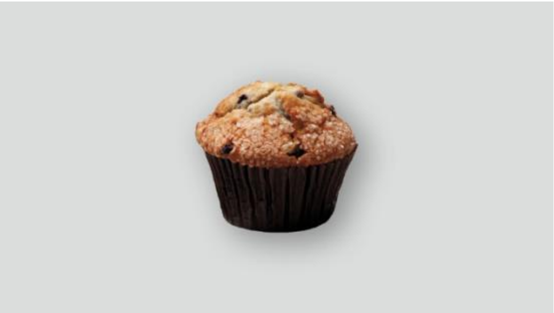

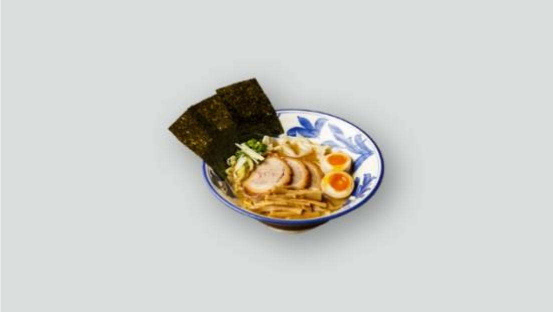
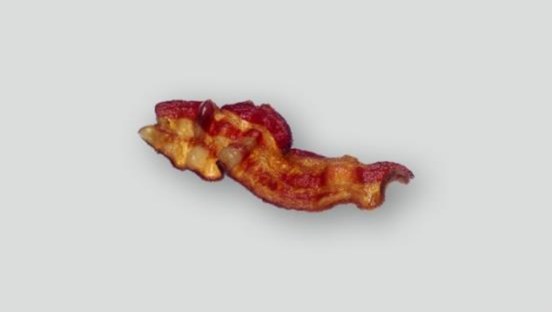
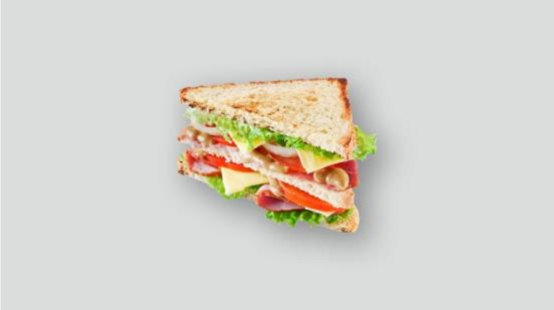
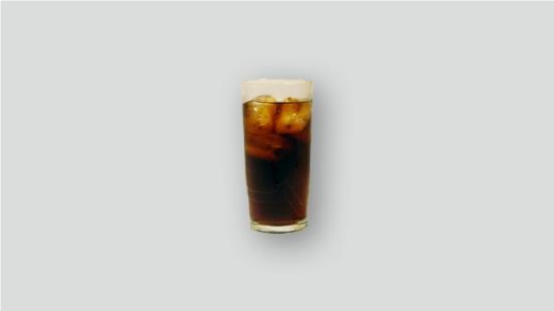
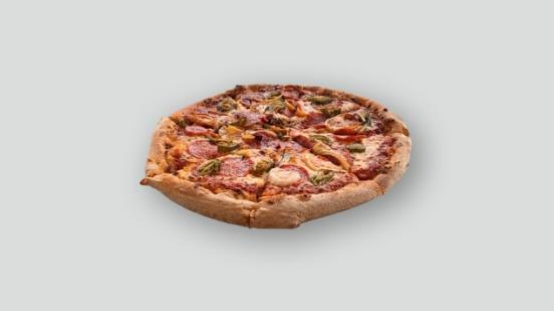
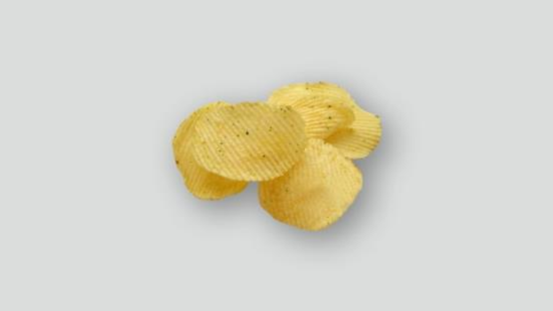
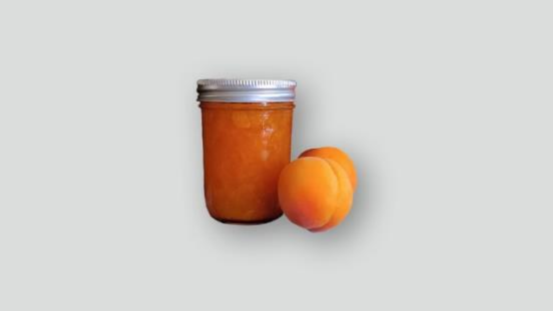
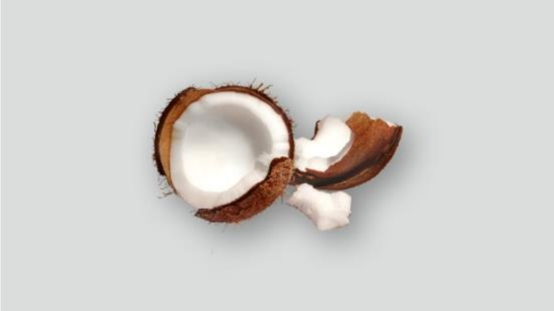
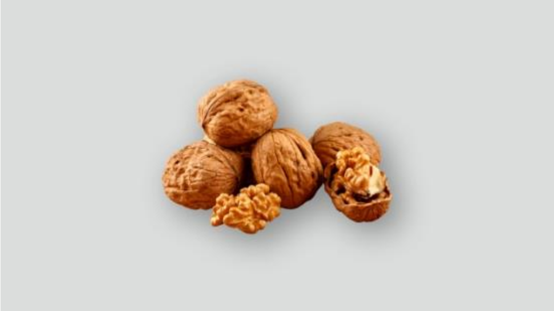
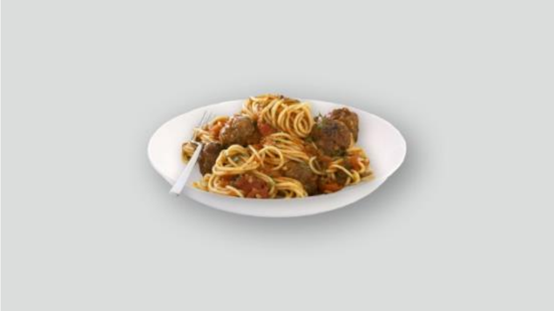

Low-calorie food stimuli

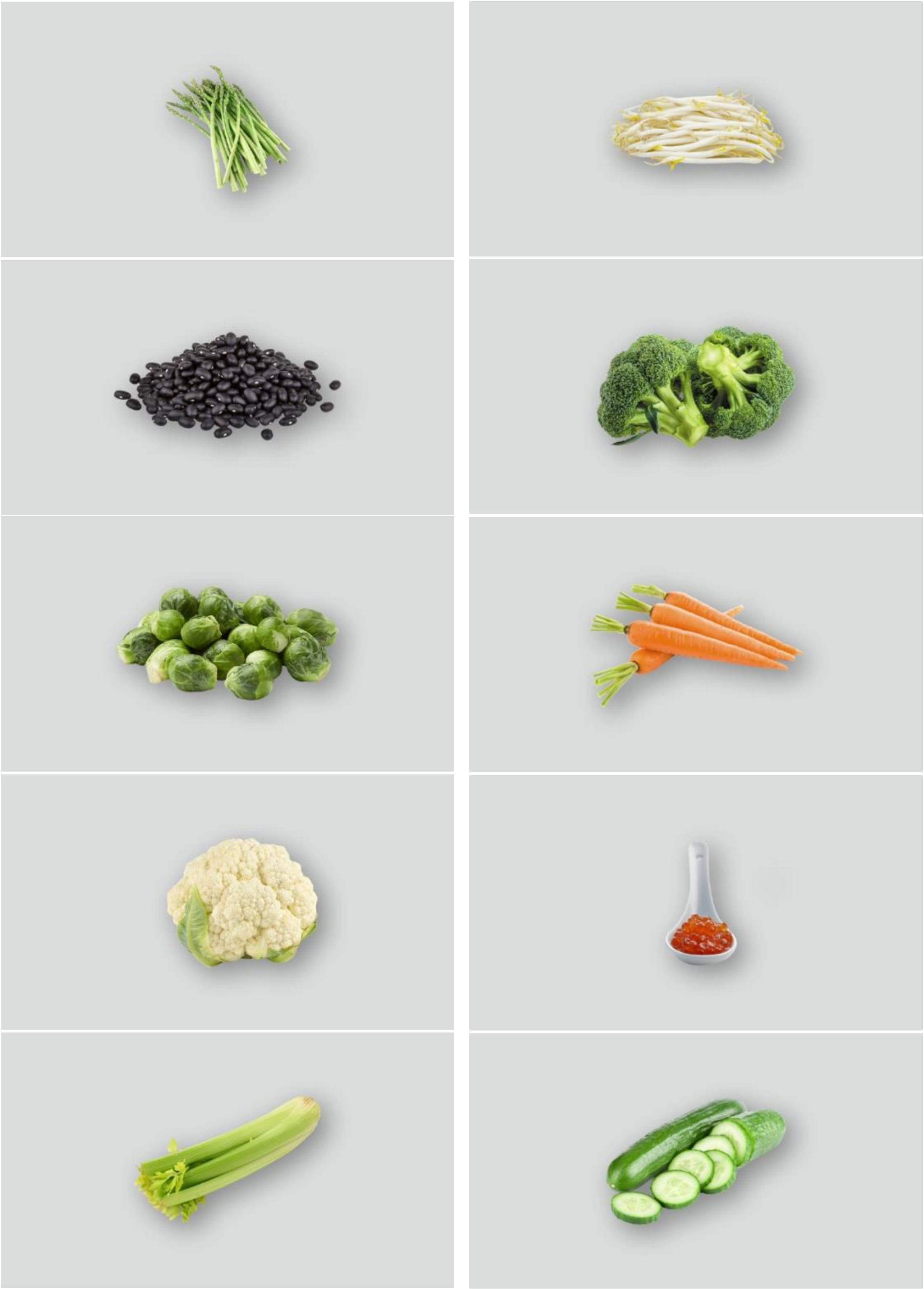

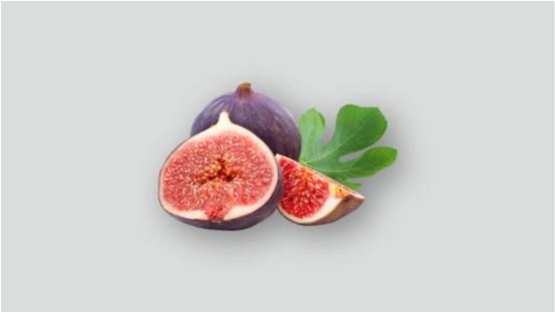
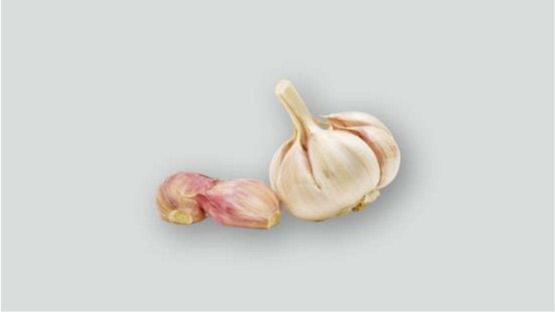
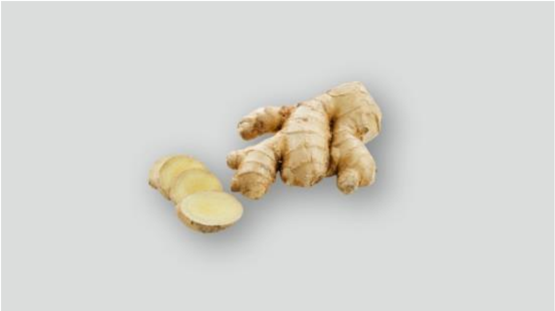
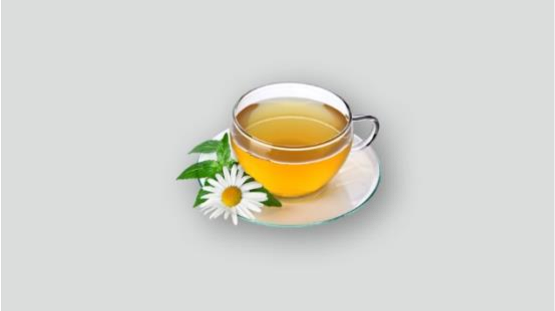
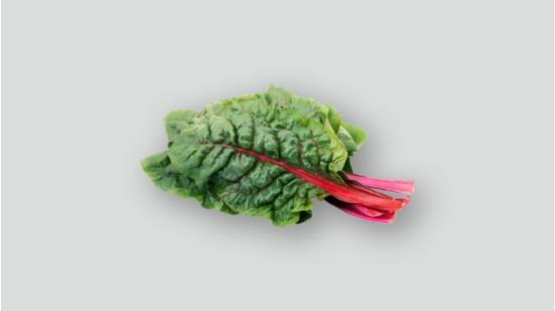
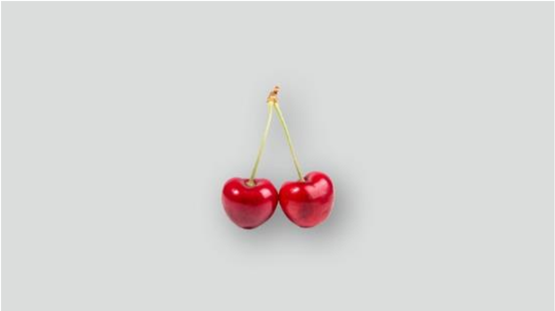
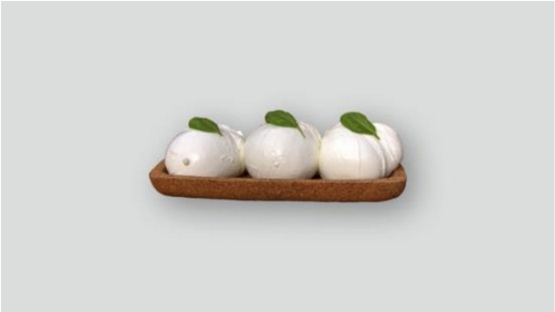
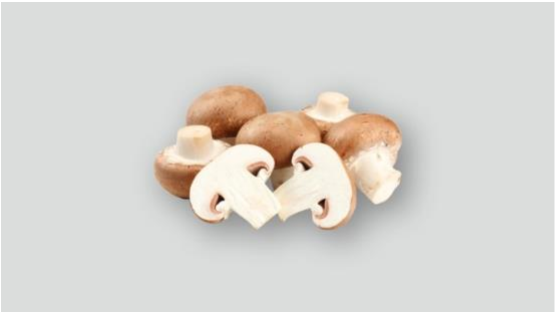
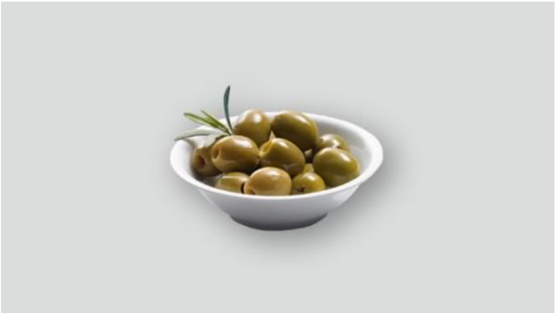
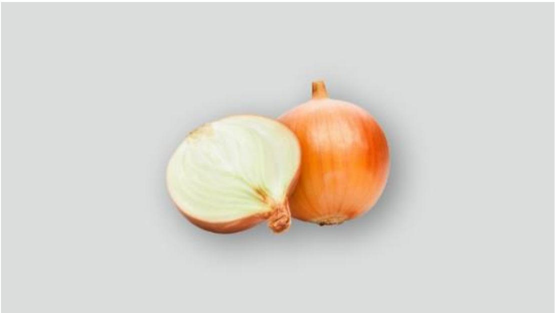

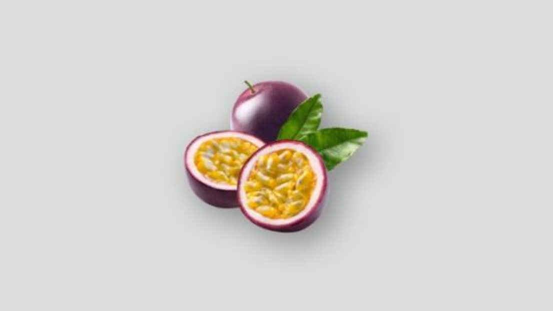
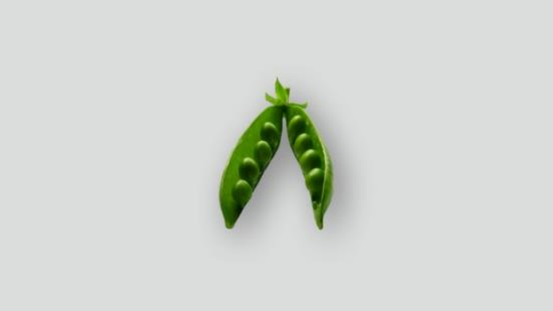
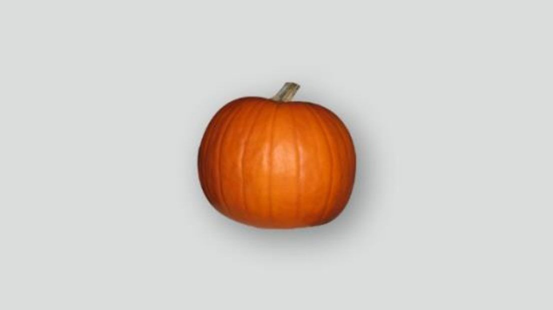
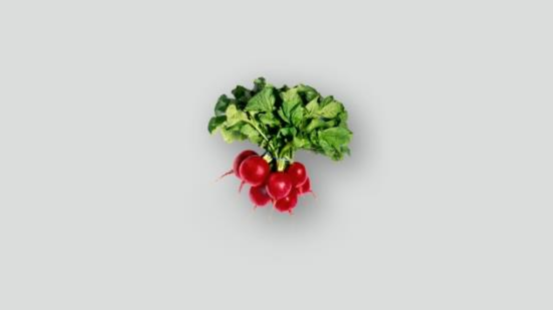
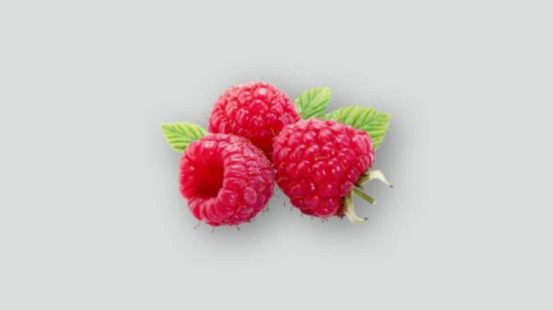
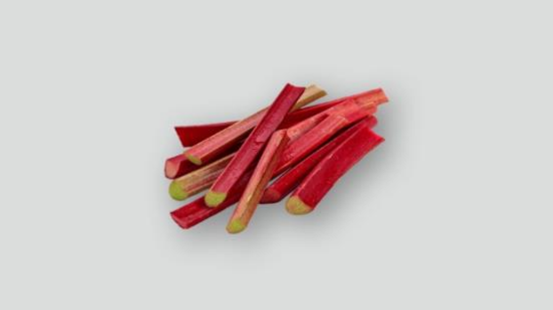
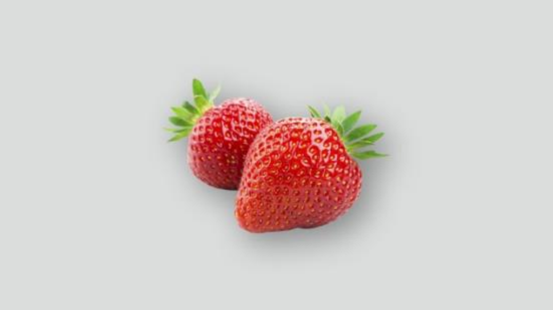
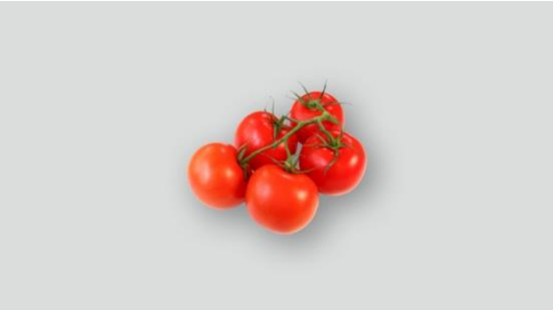
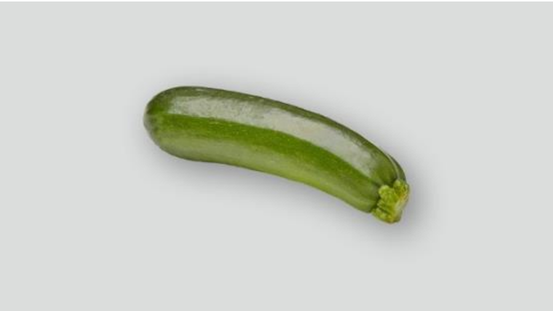
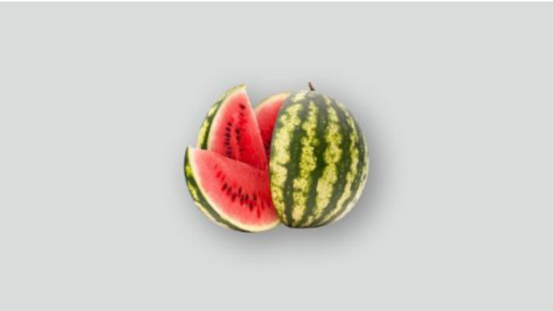

Non-food stimuli

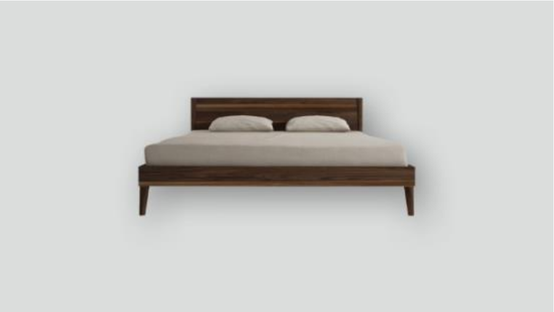
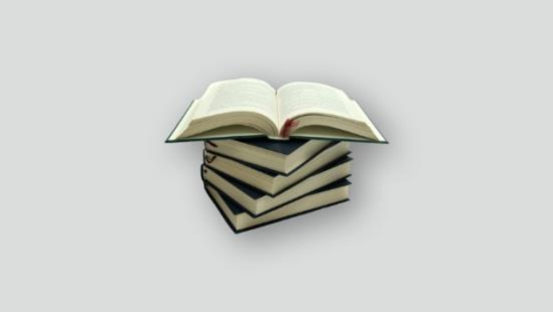
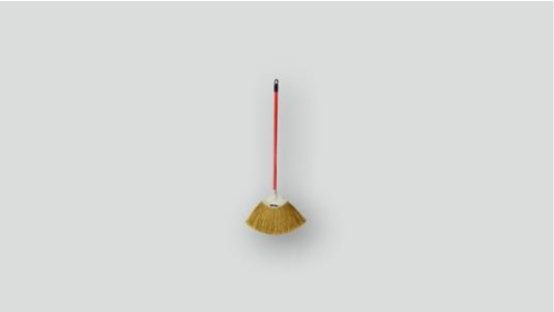
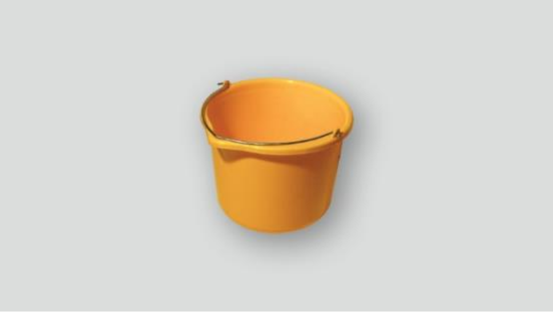
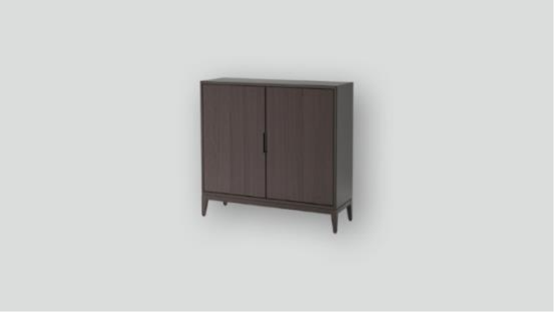
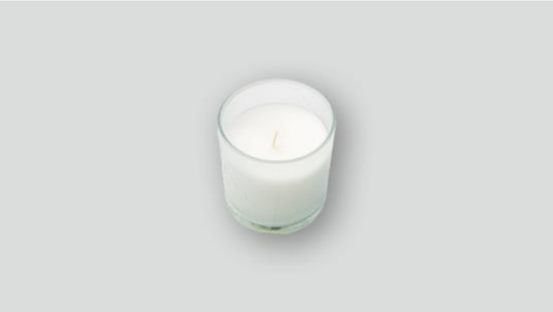
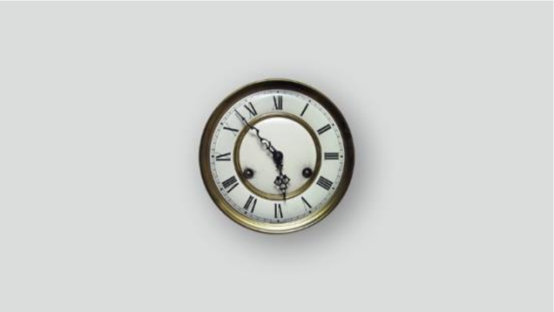
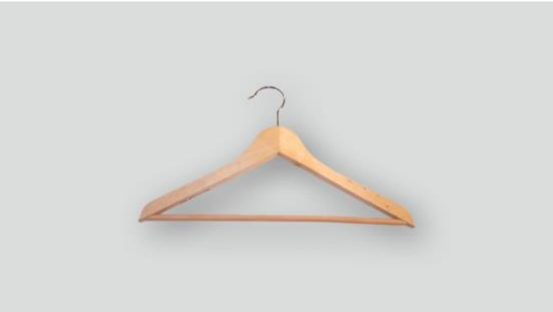
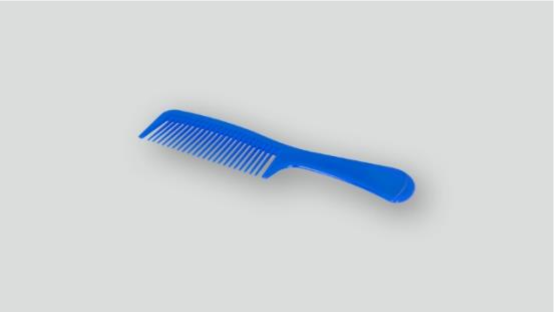
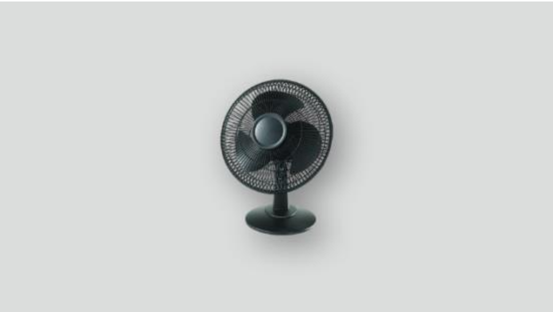

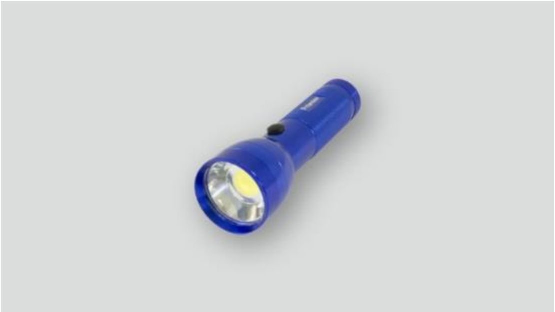
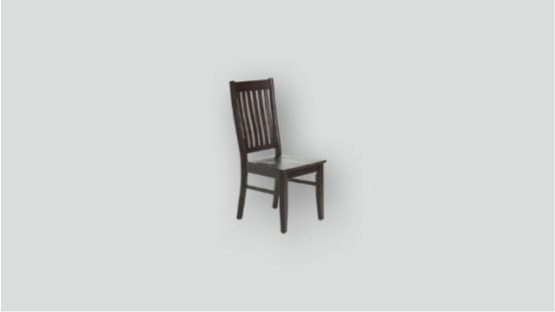
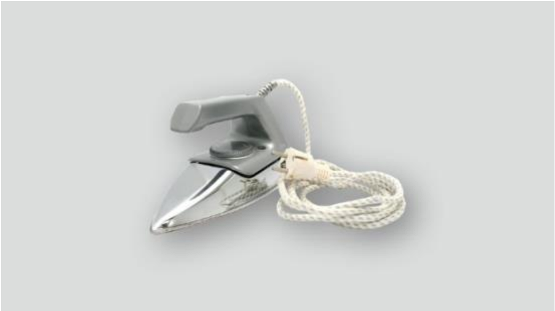
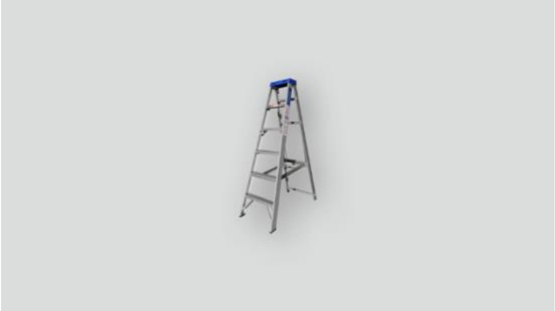
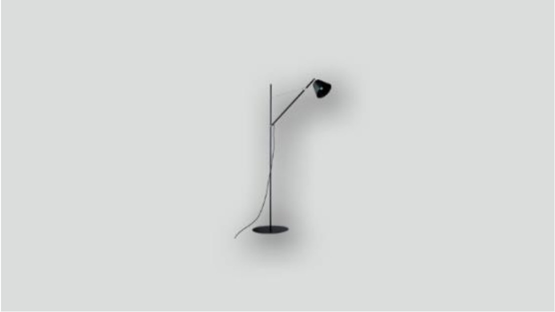
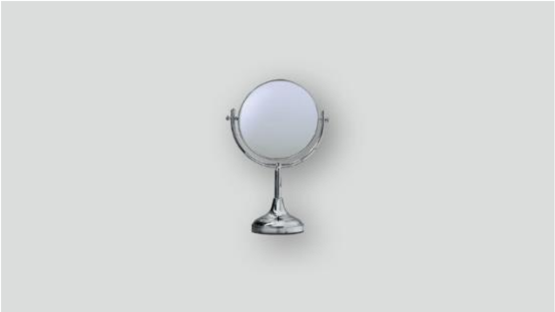
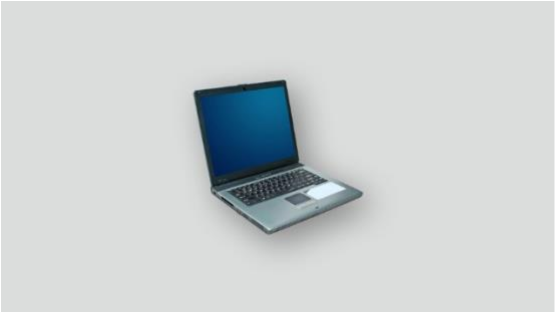
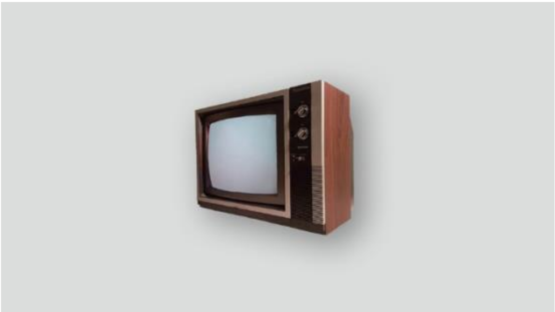
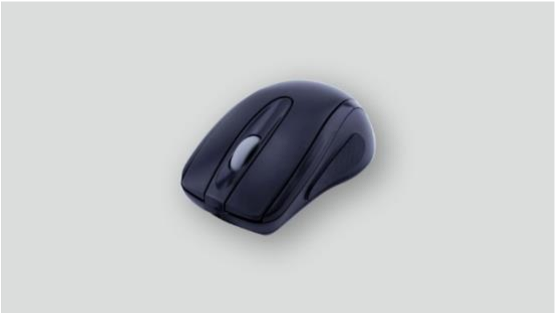
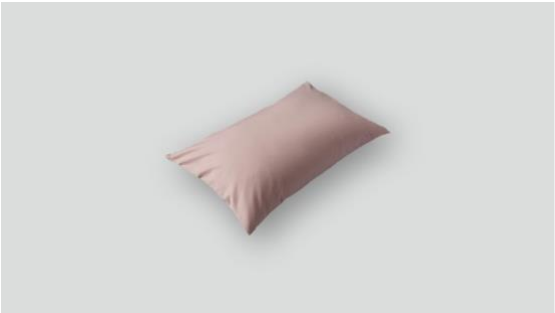

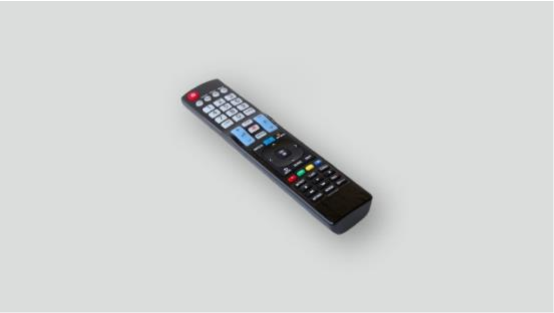
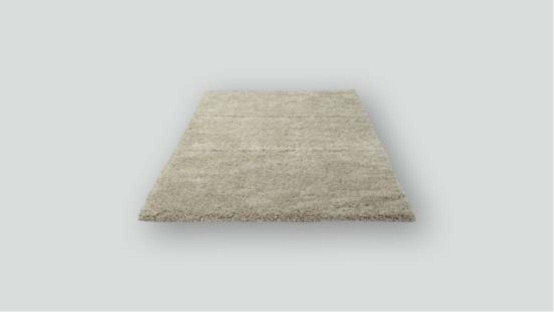
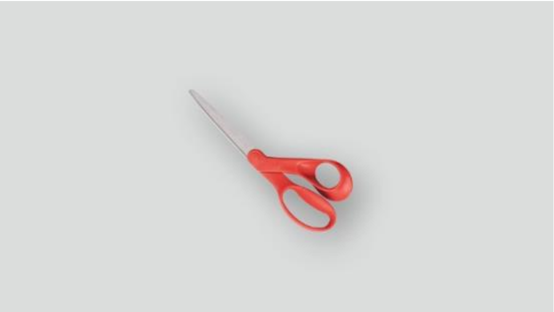
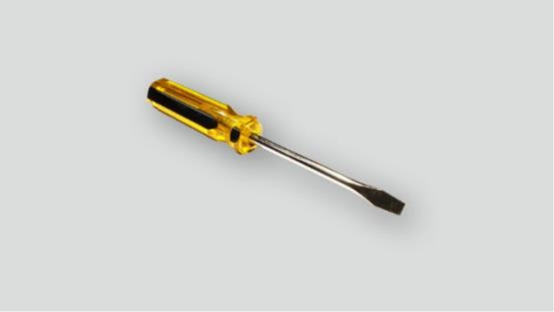
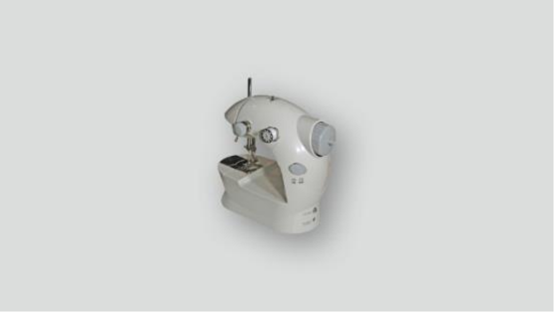
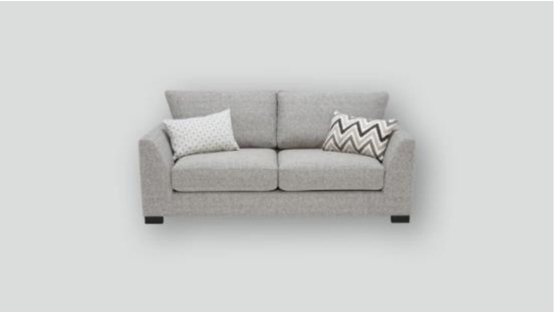
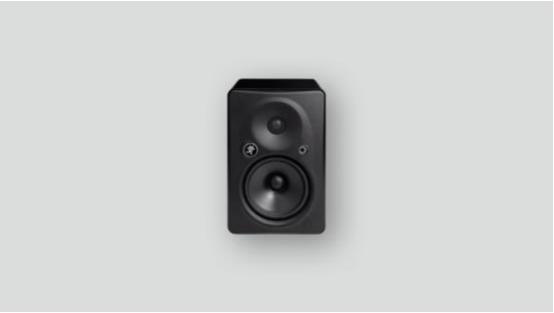
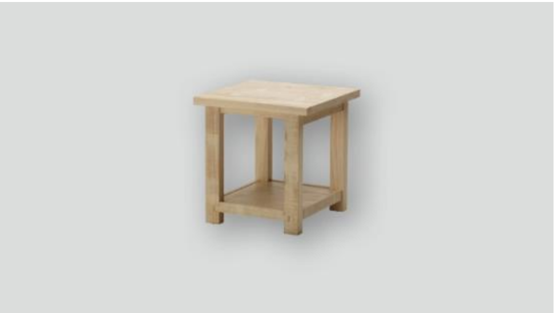
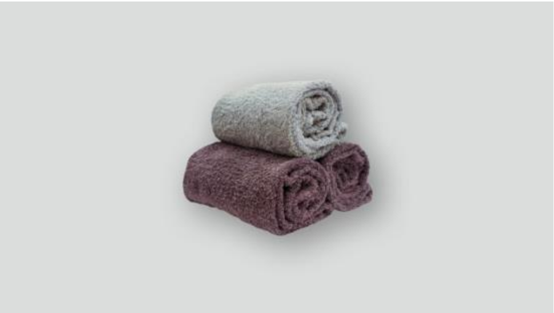
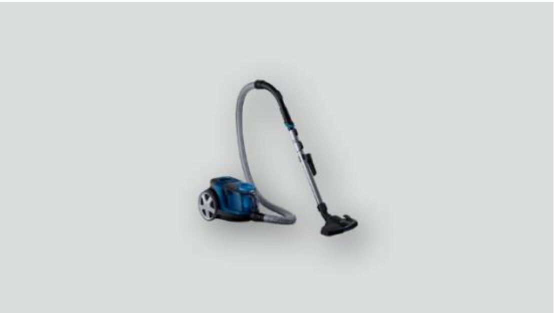

Words used in Food stroop test

			Meaning:
CELER	CELER	CELER	CELERY
ČOKOLÁDA	ČOKOLÁDA	ČOKOLÁDA	CHOCOLATE
DORT	DORT	DORT	CAKE
DVEŘE	DVEŘE	DVEŘE	DOOR
DÝNĚ	DÝNĚ	DÝNĚ	PUMPKIN
HAMBURGER	HAMBURGER	HAMBURGER	HAMBURGER
HODINY	HODINY	HODINY	CLOCK
HRANOLKY	HRANOLKY	HRANOLKY	FRENCH FRIES
HRÁŠEK	HRÁŠEK	HRÁŠEK	PEA
HRUŠKA	HRUŠKA	HRUŠKA	PEAR
JABLKO	JABLKO	JABLKO	APPLE
JAHODA	JAHODA	JAHODA	STRAWBERRY
KALENDÁŘ	KALENDÁŘ	KALENDÁŘ	CALENDAR
KNIHA	KNIHA	KNIHA	BOOK
KOBLIHA	KOBLIHA	KOBLIHA	DOUGHNUT
KOLÁČ	KOLÁČ	KOLÁČ	PIE
KOŠTĚ	KOŠTĚ	KOŠTĚ	BROOM
MELOUN	MELOUN	MELOUN	WATERMELON
MRKEV	MRKEV	MRKEV	CARROT
NŮŽKY	NŮŽKY	NŮŽKY	SCISSORS
OKNO	OKNO	OKNO	WINDOW
OKURKA	OKURKA	OKURKA	CUCUMBER
PAPÍR	PAPÍR	PAPÍR	PAPER
PAPRIKA	PAPRIKA	PAPRIKA	PEPPER
PÁREK	PÁREK	PÁREK	SAUSAGE
PIZZA	PIZZA	PIZZA	PIZZA
PRAVÍTKO	PRAVÍTKO	PRAVÍTKO	RULER
PUDING	PUDING	PUDING	PUDDING
RAJČE	RAJČE	RAJČE	TOMATO
RÝŽE	RÝŽE	RÝŽE	RICE
SLANINA	SLANINA	SLANINA	BACON
ŠPAGETY	ŠPAGETY	ŠPAGETY	SPAGHETTI
STEAK	STEAK	STEAK	STEAK
TELEFON	TELEFON	TELEFON	PHONE
TUŽKA	TUŽKA	TUŽKA	PENCIL
ŽIDLE	ŽIDLE	ŽIDLE	CHAIR
ZMRZLINA	ZMRZLINA	ZMRZLINA	ICE CREAM

:

To familiarize the participants with the procedure, a trial testing sequence was used. The order of words and their colors was the same for all participants.

ŘEKA	KALUŽ	MOŘE	OCEÁN	BYSTŘINA	PŘEHRADA	JEZERO	NÁDRŽ	POTŮČEK
Meaning:								
RIVER	PUDDLE	SEA	OCEAN	SPRING	DAM	LAKE	RESERVOIR	RIVULET
